# Bifunctional MXene‐Augmented Retinal Progenitor Cell Transplantation for Retinal Degeneration

**DOI:** 10.1002/advs.202302747

**Published:** 2023-06-28

**Authors:** Zhimin Tang, Yan Liu, Huijing Xiang, Xinyue Dai, Xiaolin Huang, Yahan Ju, Ni Ni, Rui Huang, Huiqin Gao, Jing Zhang, Xianqun Fan, Yun Su, Yu Chen, Ping Gu

**Affiliations:** ^1^ Department of Ophthalmology Shanghai Ninth People's Hospital Shanghai Jiao Tong University School of Medicine Shanghai 200011 P. R. China; ^2^ Shanghai Key Laboratory of Orbital Diseases and Ocular Oncology Shanghai 200011 P. R. China; ^3^ Materdicine Lab School of Life Sciences Shanghai University Shanghai 200444 P. R. China

**Keywords:** free radical scavenging, niobium carbide nanosheets, photothermal effect, retinal progenitor cells, retinal regeneration

## Abstract

Retinal degeneration, characterized by the progressive loss of retinal neurons, is the leading cause of incurable visual impairment. Retinal progenitor cells (RPCs)‐based transplantation can facilitate sight restoration, but the clinical efficacy of this process is compromised by the imprecise neurogenic differentiation of RPCs and undermining function of transplanted cells surrounded by severely oxidative retinal lesions. Here, it is shown that ultrathin niobium carbide (Nb_2_C) MXene enables performance enhancement of RPCs for retinal regeneration. Nb_2_C MXene with moderate photothermal effect markedly improves retinal neuronal differentiation of RPCs by activating intracellular signaling, in addition to the highly effective RPC protection by scavenging free radicals concurrently, which has been solidly evidenced by the comprehensive biomedical assessments and theoretical calculations. A dramatically increased neuronal differentiation is observed upon subretinal transplantation of MXene‐assisted RPCs into the typical retinal degeneration 10 (rd10) mice, thereby contributing to the efficient restoration of retinal architecture and visual function. The dual‐intrinsic function of MXene synergistically aids RPC transplantation, which represents an intriguing paradigm in vision‐restoration research filed, and will broaden the multifunctionality horizon of nanomedicine.

## Graphical abstract

Potent‐free radical‐scavenging niobium carbide (Nb_2_C) MXene with moderate photothermal effect and high biocompatibility enhances the performance of retinal progenitor cells (RPCs) for retinal degeneration. The synthetic Nb_2_C MXene can not only direct RPC preferentially differentiate into retinal neuronal lineages, but also substantially reduce the free‐radical levels based on systematically biological evaluations and theoretical calculations, eventually rescuing retinal structure and preserving visual function.

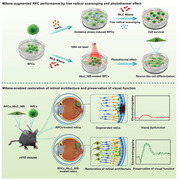

## 1 Introduction

Retinal degeneration (RD) refers to a large group of neurodegenerative diseases of the retina, which mainly include retinitis pigmentosa, macular degeneration, retinal dystrophy and diabetic retinopathy, is the most common cause of untreatable blindness.^[^ ^1^ ^]^ Although the pathogenesis of RD remains ambiguous, the majority of vision deficiency in patients with RD results from the progressive dysfunction and death of retinal neurons, especially photoreceptors.^[^ ^2^ ^]^ The mammalian retina exhibits extreme susceptibility to oxidative stress and does not have intrinsic regenerative capacity for injury repair.^[^ ^3^ ^]^

Currently available clinical therapeutics are largely palliative and cannot achieve significant visual recovery once the retinal neurons are lost. In recent years, stem/progenitor cell‐based transplantation has been considered a promising alternative for rescuing or preserving visual function by replacing and replenishing lost cells.^[^ ^4^ ^]^ Retinal progenitor cells (RPCs), as a type of donor cell, have been extensively studied in ophthalmology, which can be dissociated from the human fetal retina and can self‐renew and differentiate into multiple cell lineages.^[^ ^5^ ^]^ However, directing the RPCs to differentiate into specific retinal neuronal lineages with high accuracy, especially photoreceptors (the most crucial cell type for RD therapy), remains a predominant challenge in clinical implementation.^[^ ^6^ ^]^ Additionally, the accumulation of damaging free radicals in the pathological retinal microenvironment would undermine the viability and function of transplanted cells, and trigger continuous damage to the remaining cells and tissues, thereby leading to severely compromised clinical efficacy of RPC‐based transplantation.^[^ ^7^ ^]^ Correspondingly, a method that preferentially guides the differentiation of RPCs into retinal neurons while simultaneously ensuring the scavenging of free radicals to protect implanted donor cells in the host retina is urgently desired.

Conventional approaches, such as monolayer adhesion culture, specific molecular induction, and genetic modification have achieved some success in the neural differentiation of progenitor/stem cells.^[^ ^8^ ^]^ However, many problems, including the risk of mutation, high cost, long period of differentiation, and other unpredictable behaviors, remain to be addressed. Multiple aspects of the microenvironment, such as extracellular signals (e.g., growth factors), and intercellular signaling (e.g., exosomes and cytokines) are directly associated with the differentiation of stem cells into specific lineages.^[^ ^9^ ^]^ With advances in nanotechnology, nanomaterial‐triggered extracellular signals have shown an overwhelming advantage with respect to inducing the differentiation of stem cells into neuron‐like cells, based on the distinctive physiochemical properties of materials, such as substrate topography with optimal dimensions and geometry.^[^ ^10^ ^]^ Additionally, the precise modulation of stem cell behavior using external cues generally circumvents undesired adverse cellular events, including aberrant differentiation, overgrowth, and hyperproliferation.^[^ ^11^ ^]^

It has been reported that spatial external photothermal stimulation using near‐infrared (NIR) irradiation can tightly control the differentiation of stem cells.^[^ ^12^ ^]^ For instance, NIR‐triggered moderate photothermal therapy with nanomaterials, such as molybdenum disulfide, could precisely regulate cell differentiation with highly localized thermal effects and negligible cytotoxicity, a phenomenon that has been leveraged in bone tissue engineering.^[^ ^13^ ^]^ NIR‐induced thermal stimulation of gold‐based nanomaterials can also improve the neuronal differentiation of neural stem cells.^[^ ^14^ ^]^ However, NIR‐responsive nanomaterials with mild photothermal effect that play a positive role in directing the differentiation of RPCs into retinal neurons require further exploration. In addition, considering the fact that the normal functionalities of the implanted RPCs can be severely impaired by the accumulation of free radicals in retinal lesions, the free‐radical scavenging ability of nanomaterials is also highly emphasized, in addition to the nanostructured topography and mild photothermal therapy. Niobium carbide (Nb_2_C)—an emerging class of carbide‐based two‐dimensional (2D) MXene nanomaterials—that is photoresponsive to NIR and has the ability to form ultrathin nanostructures is gaining increasing attention in tissue engineering.^[^ ^15^ ^]^ Moreover, Nb_2_C MXene is found to be advantageous with respect to resisting oxidative stress within the tumor and osteolytic microenvironment by effectively scavenging the superoxide radicals, hydroxyl radicals, and hydrogen peroxide.^[^ ^16^ ^]^ Previous study demonstrated that Nb‐based nanoparticles exerted the neuromodulatory and neuroprotection effects on neurons by stimulating neuronal outgrowth and neurite length, which could be widely exploited for the regeneration and healing of various neuronal tissues.^[^ ^17^ ^]^ Therefore, combining the neuromodulatory and photothermal effect with antioxidative characteristics of ultrathin Nb_2_C MXene may potentially improve the performance of RPC‐based transplantation in the context of retinal regeneration.

**Scheme 1:**
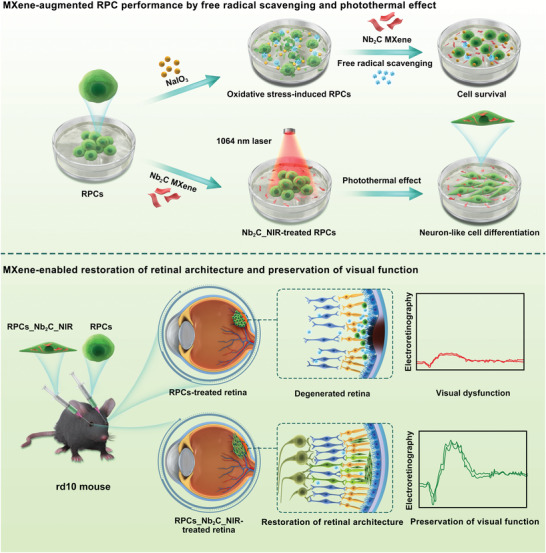
Schematic illustrating the application of MXene in RPC‐based transplantation for retinal regeneration. Upper row: Nanotherapeutic and photothermal‐conversional Nb_2_C MXene protected RPCs from NaIO_3_‐induced oxidative damage through free radical scavenging, while promoting neuronal differentiation of RPCs based on moderate photothermal stimulation. Lower row: RPCs and Nb_2_C_NIR‐treated RPCs were prepared for transplantation into the retinal degeneration 10 (rd10) mouse model. Bifunctional Nb_2_C MXene efficiently enhanced cell survival and neuronal differentiation of implanted RPCs, thereby contributing to the restoration of retinal architecture and preservation of visual function.

In this study, the cooperativity of photothermal effect and antioxidant activity in 2D Nb_2_C MXenes was first leveraged to explore the possibility of using MXene to assist RPC‐based retinal regeneration. The mechanism underlying the free‐radical elimination of Nb_2_C MXene was investigated using density functional theory calculations. Irradiation of Nb_2_C MXene with the NIR‐II biowindow (1064 nm) markedly induced the neuronal differentiation of RPCs. The potential molecular mechanism was further explored using high‐throughput RNA sequencing. Nb_2_C MXene can also synergistically serve as potent protectants for RPCs owing to its antioxidative activity. Retinal degeneration 10 (rd10) mouse with a relatively later onset and slower retinal degeneration is an eligible model to understand the pathogenesis and study the therapeutic possibilities for retinal degeneration.^[^ ^18^ ^]^ In current study, Nb_2_C MXene with NIR irradiation effectively improved the neuronal differentiation of the implanted RPCs in the host retina of rd10 mice, thereby contributing to the efficient restoration of retinal architecture and visual function (**Scheme** **1**). Together with the convenient large‐scale preparation and high biosafety, it is believed that the dual‐functional 2D MXene represents potential with respect to the future clinical management and treatment of various retinal degenerative diseases using stem cell‒based therapeutics.

## 2 Results and Discussion

### 2.1 Synthesis and Characterization of Nb_2_C MXene

**Figure 1:**
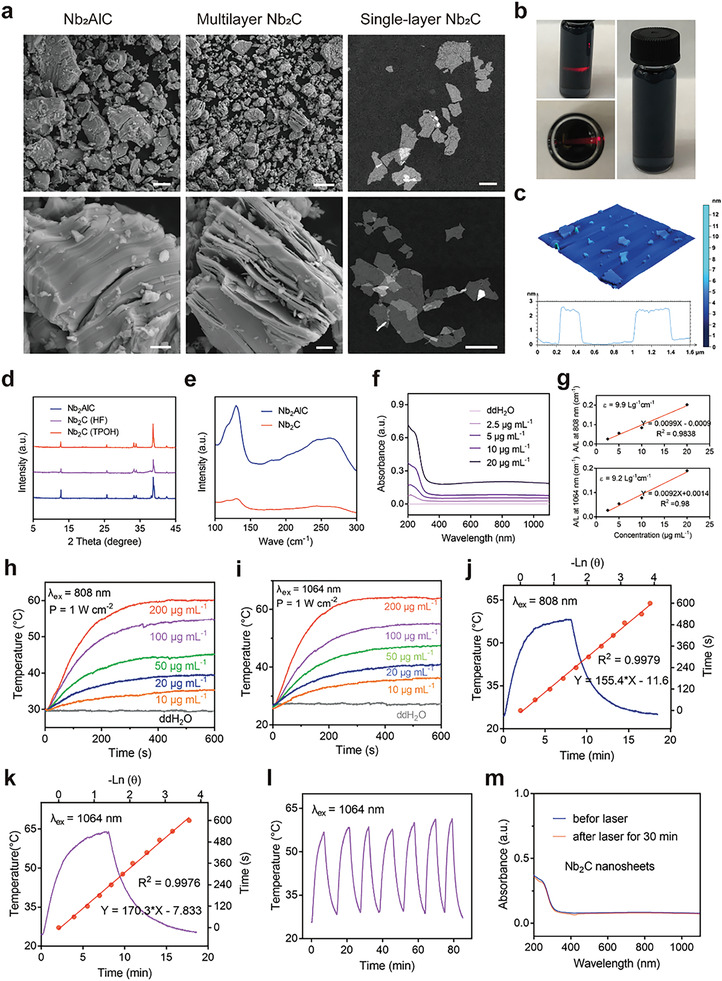
Synthesis and characterization of Nb_2_C MXene. a) SEM images of Nb_2_AlC and multilayer Nb_2_C at different magnifications, and TEM images of Nb_2_C MXene. b) Digital photographs of Nb_2_C aqueous solution. c) AFM images, and thickness distribution analysis of Nb_2_C MXene. d) XRD profiles of Nb_2_AlC, multilayer Nb_2_C, and Nb_2_C MXene. e) Raman spectra of Nb_2_AlC and Nb_2_C MXene. f) UV–vis spectra of various concentrations of Nb_2_C MXene. g) The mass extinction coefficient of Nb_2_C MXene at 808 and 1064 nm. Temperature elevation curves of different doses of Nb_2_C MXene under h) 808 nm and i) 1064 nm laser exposure (1 W cm^−2^). Photothermal profile of Nb_2_C aqueous solution under j) 808 nm and k) 1064 lasers and the corresponding linear relationship between time and logarithm of the temperature changes during the cooling process. l) Photothermal curves of Nb_2_C aqueous solution for six on/off irradiation cycles by a 1064 nm laser (1 W cm^−2^). m) UV–vis spectra of Nb_2_C MXene before and after irradiation by a 1064 nm laser for 30 min.

2D Nb_2_C MXene was fabricated using a chemical exfoliation strategy in accordance with a previously reported method.^[^ ^19^ ^]^ First, the Al layer of bulk Nb_2_AlC was etched using aqueous hydrofluoric acid (HF) aqueous solution. The multilayer structure of Nb_2_C powder was observed using scanning electron microscopy (SEM) (**Figure** **1**a). Further delamination in tetrapropylammonium hydroxide (TPAOH) facilitated the fabrication of ultrathin Nb_2_C MXene. Transmission electron microscopy (TEM) confirmed the successful formation of ultrathin Nb_2_C MXene with planar sizes ranging from several to hundreds of nanometers (Figure 1a). Figure 1b shows representative digital photographs of bulk Nb_2_C. In addition, the thickness of the Nb_2_C MXene was determined to be 0–2.5 nm, which was in conformity with the findings in our previous report (Figure 1c).^[^ ^19^ ^]^ As evidenced by the X‐ray diffraction (XRD) patterns and Raman spectra of Nb_2_AlC and Nb_2_C MXene, the bulk Nb_2_AlC was completely converted to 2D ultrathin MXene upon using the HF etching and the TPAOH intercalation approach (Figure 1d,e). Therefore, the obtained Nb_2_C MXene was characterized as ultrathin nanostructures, thereby facilitating in vitro and in vivo biomedical applications.

### 2.2 Photothermal Conversion Performance and Free‐Radical Scavenging Capability of Nb_2_C MXene

The as‐prepared Nb_2_C MXene exhibited intense and broad NIR absorbance ranging from 600 to 1200 nm. Their mass extinction coefficient at 808 and 1064 nm was found to be 9.9 and 9.2 L g^−1^ cm^−1^, respectively (Figure 1f,g), which was evidently higher than reported photothermal conversion agents, such as gold nanorods (3.9 L g^−1^ cm^−1^) and graphene oxide (3.6 L g^−1^ cm^−1^).^[^ ^20^ ^]^ We further explored their photothermal conversion performance using 808 and 1064 nm lasers, which are indispensable for the construction of desirable photothermal conversion agents. The temperature elevation of Nb_2_C at various doses under continuous laser exposure was recorded using an infrared thermal‐imaging system. The temperature rise exhibited peculiar irradiation time‐ and concentration‐dependent patterns (Figure 1h,i; Figures S1–S4, Supporting Information). When the Nb_2_C dose was increased from 10 to 200 µg mL^−1^, the solution temperature promptly increased by 5 to 40 °C after irradiation with an 808 nm laser for 10 min (Figure 1h). In contrast, a negligible temperature increase was observed in deionized water under identical experimental conditions. Additionally, the temperature increase could also be regulated from 20 °C (0.5 W cm^−2^) to 45 °C (1.5 W cm^−2^) by adjusting the power density of the applied 808 nm laser (Figure S3, Supporting Information). The collective results demonstrated that as‐prepared Nb_2_C MXene can achieve efficient conversion of laser to thermal energy. The photothermal conversion efficiency of Nb_2_C under conditions of 808 and 1064 nm laser irradiation was 35.6% and 42.8%, respectively (Figure 1j,k). The photothermal stability of the Nb_2_C MXene was investigated by monitoring the temperature changes of the aqueous Nb_2_C solutions under several alternating heating and cooling processes when irradiated with 808 and 1064 nm laser irradiation (Figure 1l,m; Figure S5, Supporting Information). Negligible temperature fluctuations were observed during the observation period, indicating that Nb_2_C MXene can serve as a durable photothermal conversion agent for stem cell‐based regenerative therapy.

**Figure 2:**
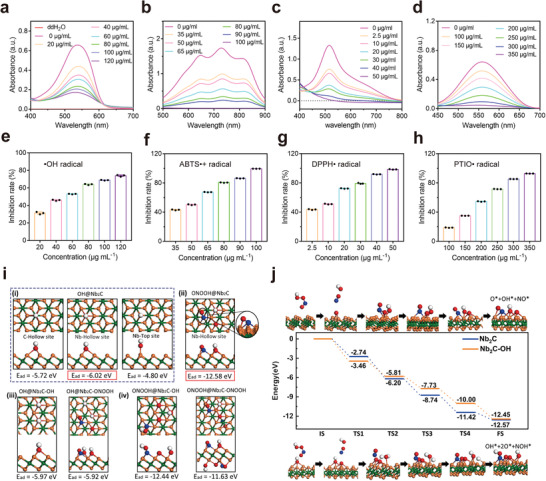
Free‐radical scavenging capability of Nb_2_C MXene. ROS and RNS scavenging performance of Nb_2_C MXene for a,e) •OH, b,f) ABTS, c,g) DPPH, and d,h) PTIO, *n* = 3, data presented as mean ± SD. i) Adsorption energies of i) ROS (•OH) and ii) protonated RNS (ONOOH) on Nb_2_C MXene together with that of iii) •OH and iv) ONOOH on Nb_2_C MXene after adsorption of •OH and ONOOH. j) Energy drop diagram of the decomposition process of ONOOH on pure Nb_2_C MXene (top row) and Nb_2_C MXene after adsorption of •OH (bottom row). The green, orange, red, blue, and white balls indicate C, Nb, O, N, and H atoms, respectively.

The scavenging efficiencies of reactive oxygen species (ROS) and reactive nitrogen species (RNS) were assessed. High efficiency and dose‐dependent scavenging of hydroxyl radicals (•OH) were detected, demonstrating the peroxidase‐like activity of the designed Nb_2_C MXene (**Figure** **2**a,e). For instance, ≈75% of •OH was eliminated by Nb_2_C MXene at a concentration of 100 µg mL^−1^. In addition to ROS, the overproduction of RNS, including ONOO^−^ and •NO, can also induce inflammation, DNA damage, and cell death.^[^ ^21^ ^]^ Therefore, the RNS‐eliminating capability of the Nb_2_C MXene was also investigated using 2,2′‐azino‐bis(3‐ethylbenzothiazoline‐6‐sulfonic acid) (ABTS), 2,2‐di‐(4‐tert‐octylphenyl)‐1‐picrylhydrazyl (DPPH), and 2‐phenyl‐4,4,5,5‐tetramethylimidazoline‐3‐oxide‐1‐oxyl (PTIO). ABTS, DPPH, and PTIO were efficiently and completely eliminated at doses of 100, 50, and 350 µg mL^−1^, respectively (Figure 2b–d,f–h), demonstrating the high RNS scavenging efficiency of Nb_2_C MXene. All the results suggested the excellent capability of Nb_2_C MXene to scavenge ROS and RNS.

To evaluate the mechanism underlying the scavenging of ROS and RNS, we performed density functional theory calculations using the Vienna Ab initio Simulation Package on the adsorption progress of •OH and ONOOH (protonated ONOO^−^) on Nb_2_C MXene. First, the adsorption sites of C‐Hollow, Nb‐Hollow, Nb‐Top, and Nb‐bridge of a typical ROS molecule •OH were compared to identify the most stable adsorption site. Their optimized atomic structures were illustrated as model (i) in Figure 2i. The adsorption energy was defined by the following equation

(1) $$E_{\mathrm{ad}}=E_{\mathrm{system}}-E_{\mathrm{Nb}2C\;\mathrm{substrate}}-E_{\mathrm{molecule}}$$

where *E*_system_ and *E*_Nb2C subatrate_ are total energies of the whole molecule@Nb_2_C system and the substrate before adsorption of ROS or RNS molecules, respectively, and *E*_molecule_ is the energy of the adsorbed molecule. According to this definition, the more negative *E*_ad_ more stable is the system. The adsorption energy of the model with Nb‐Hollow site was calculated to be −6.02 eV, 0.3 eV (1.22 eV) lower than that of the model with C‐Hollow (Nb‐Top) site. The values explain the theoretical possibility for the observed experimental ROS scavenging. The model featuring the Nb‐bridge site also tended to form an Nb‐Hollow site after the structural optimization (Video S1, Supporting Information). This finding illustrated that the adsorption model with the Nb‐Hollow site is the energetically favorable one. Concerning the scavenging of ONOOH, it decomposed into OH*, O*, and ON* adsorbed on the Nb_2_C surface with a much lower adsorption energy of −12.58 eV (model (ii) in Figure 2i), demonstrating that ONOO^−^ is easily scavenged by Nb_2_C MXene. Next, we investigated the effect of adsorbed molecules on the ROS scavenging ability of Nb_2_C MXene, as shown in model (iii) in Figure 2i. The observed adsorption energies of •OH on Nb_2_C MXene adsorbed with •OH and ONOOH were −5.97 and −5.92 eV, respectively, which approximated the adsorption energy on pure Nb_2_C MXene (−6.02 eV). These findings demonstrated that the adsorbed ONOOH and •OH species does not impact the subsequent ROS scavenging process. Similarly, the adsorption energies of ONOOH on Nb_2_C MXene adsorbed with ONOOH and •OH were −11.63 and −12.44 eV, respectively (model (iv) in Figure 2i), which were very close to the adsorption energy on pure Nb_2_C MXene (−12.58 eV). Therefore, the Nb_2_C MXene could simultaneously scavenge both ROS and RNS (together referred to as RONS). Additionally, the surface RONS scavenging capacity of the Nb_2_C MXene remained almost constant until the adsorption capacity was saturated.

To further explain the influence of OH* on ONOOH decomposition, we calculated the energy profile of the process with or without OH* on the surface. The initial state was set as ONOOH away from the MXene (Figure 2j). Without OH*, ONOOH adsorbed on the surface through the fracture of O—N, O—O bonds, together with the formation of O—Nb bonds, resulting in an energy drop of −2.74 eV. Then, it went through several transition states (TSs) accompanied by energy drops of −3.46, −2.54, −2.68, and −1.15 eV, and formed O* and OH* species at the most stable Nb‐Hollow sites, as well as NO* (N is also located at Nb‐Hollow site) on the nanosheet surface. The total energy drop during this process was −12.57 eV, demonstrating that the adsorption and decomposition of ONOOH on the pure Nb_2_C surface were energetically favorable. For Nb_2_C MXene adsorbed with OH*, ONOOH adsorbed on the surface with a higher energy drop (−3.46 eV) and eventually formed OH*, NO*, and two O* on the surface after several TSs with energy drops of −2.35, −1.92, −2.27, and −2.45 eV, representing a total cumulative energy drop of −12.45 eV. Their similar energy profiles further confirmed that the presence of OH* does not damage the ONOOH adsorption and decomposition process.

### 2.3 In Vitro and In Vivo Biocompatibility of Nb_2_C MXene

**Figure 3:**
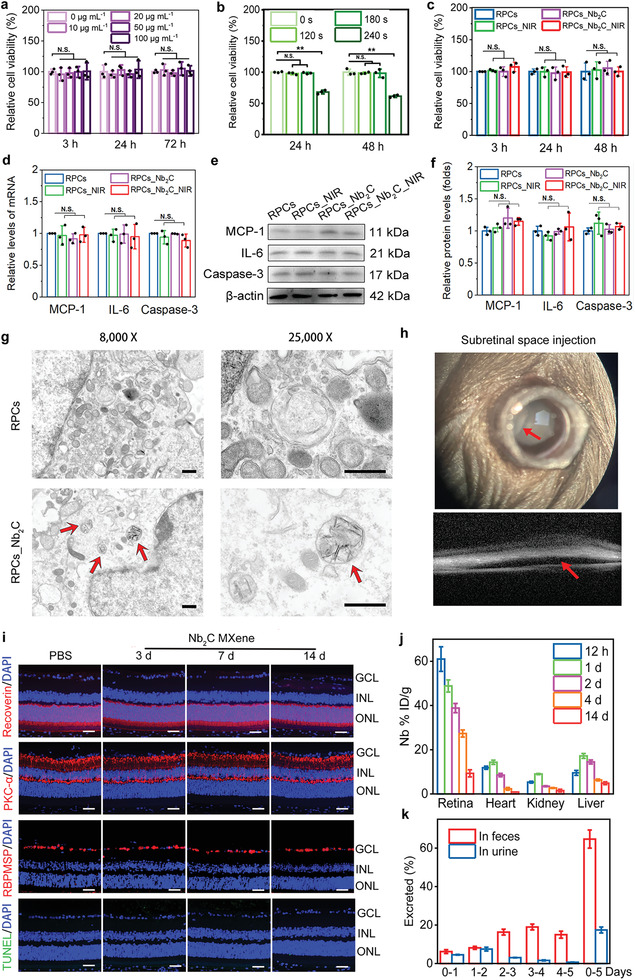
In vitro and in vivo biocompatibility of Nb_2_C MXene. Effects of a) Nb_2_C MXene at different dose and b) Nb_2_C MXene (50 µg mL^−1^) with NIR‐II irradiation (1064 nm, 1 W cm^−2^) for different time (120, 180, and 240 s) on cell viability were determined using CCK‐8 assay. *n* = 3, one‐way ANOVA with Bonferroni correction, not significant (N.S.) >0.05, data presented as mean ± SD. c) Effects of NIR‐II (1064 nm, 1 W cm^−2^, 180 s), Nb_2_C (50 µg mL^−1^), and Nb_2_C_NIR on cell viability were evaluated after treatment for 3, 24, and 48 h. *n* = 3, one‐way ANOVA with Bonferroni correction, not significant (N.S.) > 0.05, data presented as mean ± SD. Effects of NIR, Nb_2_C, and Nb_2_C_NIR on cell inflammatory indicators (MCP‐1 and IL‐6) and apoptosis biomarker Caspase‐3 were determined after treatment for 24 h by evaluating d) gene and e,f) protein expression levels. *n* = 3, one‐way ANOVA with Bonferroni correction, not significant (N.S.) > 0.05, data presented as mean ± SD. g) Cellular internalization of Nb_2_C MXene was imaged utilizing TEM. Red arrows: Nb_2_C‐encapsulated organelle. Scale bars: 500 nm. h) Representative photos of subretinal injection of Nb_2_C MXene into the healthy mice. Red arrow, a bleb at the injected site. i) Detection of Recoverin‐ (red), PKC‐*α*‐ (red), RBPMS‐ (red), and TUNEL‐ (green) positive retinal cells in healthy mice 3 d, 7 d, and 14 d after treatment with Nb_2_C MXene. Cell nuclei were counterstained with DAPI (blue). GCL: ganglion cell layer; INL: inner nuclear layer; ONL: outer nuclear layer. Scale bar: 50 µm. j) Biodistribution of Nb (% injected dose (ID) of Nb per gram of tissue) in retina, heart, kidney, and liver after subretinal injection of 1 mg mL^−1^ Nb_2_C MXene for 12 h, 1 d, 2 d, 4 d, and 14 d, *n* = 3, data presented as mean ± SD. k) Excretion of Nb from urine and feces after the injection of Nb_2_C MXene over 5 days. *n* = 3, data presented as mean ± SD.

To identify whether the primarily isolated RPCs used in present study are pure, we investigated the expression levels of neural progenitor‐related markers, including Pax‐6, Nestin, and Vimentin. As presented in Figure S6a,b (Supporting Information), we found a high positive ratio of Pax‐6‐ (97.3%), Nestin‐ (95.7%), and Vimentin‐ (97.2%) expressed cells in the isolated cells, but glial cell marker GFAP‐positive cells were undetectable upon immunocytochemistry staining. The result indicated that the separated cells are almost pure RPCs, which is consistent with our previous studies.^[^ ^22^ ^]^ The in vitro cytotoxicity of nanomaterials must be carefully addressed prior to their in vivo exposure and accumulation. With this aim, we initially explored the cellular biocompatibility of Nb_2_C MXene using CCK‐8 analysis. Nb_2_C administration at different concentrations (10, 20, 50, and 100 µg mL^−1^) for 3 h (*p* = 0.562, *p* = 0.691, *p* = 0.949, and *p* = 0.934, respectively), 24 h (*p* = 0.589, *p* = 0.733, *p* = 0.609, and *p* = 0.742, respectively), and 72 h (*p* = 0.655, *p* = 0.48, *p* = 0.472, and *p* = 0.79, respectively) produced no obvious detriments on cellular viability (**Figure** **3**a). To optimally prevent cell damage and avoid molecular perturbation, a final concentration of 50 µg mL^−1^ was chosen for subsequent experiments.^[^ ^16a^ ^]^ The high biosafety of nanoparticles (50 µg mL^−1^) was additionally confirmed by live/dead staining (Figure S7a,b, Supporting Information). To further determine the photothermal effect of Nb_2_C on cytocompatibility, NIR‐II (1064 nm) irradiation using a 1.0 W cm^−2^ laser was performed once per day to irradiate the RPCs for 120, 180, and 240 s. When the duration of laser irradiation was less than 180 s and then cultured for 24 h (*p* = 0.067 for 120 s, and *p* = 0.164 for 180 s), and 48 h (*p* = 0.656 for 120 s, and *p* = 0.738 for 180 s), the cell viability remained higher than 95%, but decreased at the extended irradiation time (240 s, *p* < 0.001) (Figure 3b). Mild heat stimulation at 38–40 °C is considered to be biosafe and beneficial for neuronal differentiation.^[^ ^23^ ^]^ Therefore, according to the temperature elevation curves depicted in Figure 1i, NIR‐II irradiation for 180 s with a 1.0 W cm^−2^ laser on Nb_2_C MXene (50 µg mL^−1^) was found to be optimal and was performed for all subsequent bexperiments. Furthermore, no marked difference in cell viability was observed between the control and treated cells, irrespective of the presence of Nb_2_C MXene with or without NIR‐II modulation (Figure 3c). To better illustrate the impact of Nb_2_C, NIR, and Nb_2_C_NIR on cell inflammatory and apoptotic responses, the mRNA expression levels of monocyte chemoattractant protein‐1 (MCP‐1, an intraocular inflammation‐related marker), interleukin‐6 (IL‐6, which is involved in pro‐inflammatory progress), and Caspase‐3 (a key apoptotic indicator) were evaluated.^[^ ^22^ ^]^ As shown in Figure 3d–f, the gene and protein expression levels of MCP‐1, IL‐6 and Caspase‐3 in untreated RPCs were similar to those in NIR‐, Nb_2_C‐, and Nb_2_C_NIR‐treated RPCs. These findings indicated that Nb_2_C treatment elicited negligible inflammatory and apoptotic responses, regardless of NIR irradiation. Theoretically, Nb_2_C MXene can be internalized by RPCs, owing to their ultrathin 2D nanostructures.^[^ ^24^ ^]^ Indeed, bright‐field TEM imaging revealed the substantial encapsulation of Nb_2_C MXene into RPCs (marked by red arrows) without disruption of intracellular organelles (Figure 3g). These observations verified the high cytocompatibility of Nb_2_C MXene, laying a solid foundation for efforts to guide the neuronal differentiation of RPCs.

One of the unique features of nanomaterials is their relatively slower metabolism in the host than that of low‐molecular‐weight compounds. Consequently, nanomaterials possess long‐term in vivo effects, while also probably triggering unwanted side effects.^[^ ^25^ ^]^ Therefore, the biodistribution, clearance, and host tissue response profiles against Nb_2_C MXene are not only critical for determining its biosafety, but also helpful in identifying the in vivo therapeutic effects of the MXene. To observe the in vivo biocompatibility of Nb_2_C MXene, TUNEL staining and immunofluorescence (IF) staining of retinal sections were conducted at days 3, 7, and 14 after a single injection of Nb_2_C MXene into the subretinal space of healthy C57BL/6 mice. It is challenge to perform subretinal injection in mice. Thus, the outcome of the subretinal injection was determined by spectral domain optical coherence tomography (SD‐OCT). As illustrated in Figure 3h, a successful subretinal injection of Nb_2_C MXene (1 µL per eye, 1 mg mL^−1^) into healthy C57BL/6 mice was confirmed by the formation of a bleb at the injected site (red arrow). Similar to the phosphate‐buffered saline (PBS) injection, the TUNEL‐positive cells were negligible in all retinal layers of retinae treated with Nb_2_C MXene at different period, and the unchanged protein expression levels of Recoverin (a marker of photoreceptor cells), PKC‐*α* (a marker of bipolar cells), and RBPMS (a marker of ganglion cells) were observed in different retinal layers at day 3, 7 and 14 postadministration of Nb_2_C MXene (Figure 3i). Additionally, retinal structure and thickness were nearly unchanged after exposure to Nb_2_C MXene in comparison to the PBS‐treated retinae. Additionally, hematoxylin and eosin staining (H&E) staining of the tissues collected from mice treated with Nb_2_C MXene revealed the absence of obvious pathological effects on the main organs, including the heart, kidney, spleen, liver, and lung, compared to the PBS‐treated control group 14‐day postadministration (Figure S8, Supporting Information). These results preliminarily indicated that Nb_2_C MXene exhibits excellent in vitro and in vivo biocompatibility, with a reasonable biodegradation profile for retinal regeneration.

It has been reported that Nb_2_C‐PVP after intravenous administration distributed in almost all tissues, such as liver, heart, kidney of BALB/C mice, and could be metabolized efficiently in these tissues with the detection of Nb element (as the degradation product of Nb_2_C‐PVP).^[^ ^16a^ ^]^ To investigate whether the Nb_2_C MXene (1 µL per eye, 1 mg mL^−1^) after a single subretinal injection into healthy C57BL/6 mice can be taken up by the host's tissues, and then degraded in the body, the Nb element in main organs (including retina, heart, liver, and kidney), as well as urine and feces samples was analyzed by ICP‐AES. As shown in Figure 3j, 12 hours (12 h) postinjection, a large amount Nb_2_C MXene (60% of injected dose (ID)/g (tissues)) existed in the retina, and a similar uptake in liver, heart, and kidney was observed. One day (1 d) after Nb_2_C MXene administration, the maximum accumulation of Nb in liver, heart, and kidney was found with 17.2%, 14.33%, and 9% of ID/g (tissues), respectively, and Nb content decreased with time after that. Especially, at day 14 after subretinal administration, residual Nb in the retina, heart, kidney and liver was decreased to 9.33%, 0.85%, 1.5%, and 4.87% of ID/g (tissues), respectively, indicating that Nb_2_C MXene can be scavenged in different organs. Additionally, ≈82.15% of Nb could be excreted through feces (64.7%) and urine (17.45%) over 5 days postinjection (Figure 3k). These results suggested that subretinally injected Nb_2_C MXene could exert bioeffects on retina for at least 14 days, and the Nb_2_C MXene may be released by retina, then entered to various tissues via blood circulation, and finally be cleared through the urine and feces, which is similar to the results of Nb_2_C‐PVP with intravenous injection into BALB/C mice.^[^ ^16a^ ^]^ Collectively, the implanted Nb_2_C MXene possessed a favorable in vivo biosafty profile and an unique characteristic of clearance by the body.

### 2.4 Nanoprotection of Oxidative Stress‐Induced RPCs by Nb_2_C MXene

**Figure 4:**
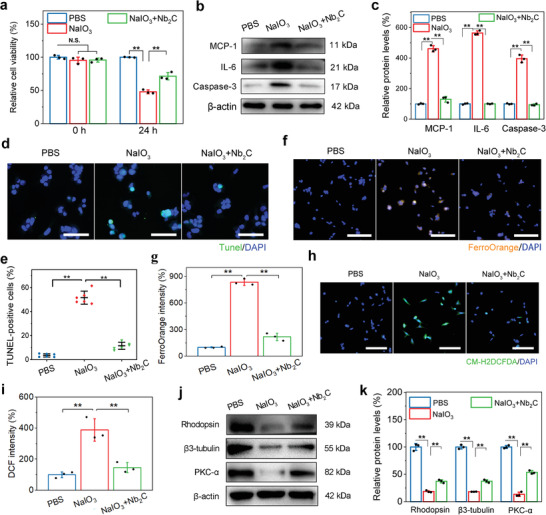
Nanoprotection of oxidative stress‐induced RPCs by Nb_2_C MXene. Three groups including RPCs treated with PBS, NaIO_3_ (10 × 10^−3^ m), and NaIO_3_ + Nb_2_C MXene under differentiation condition, 24 h later, a) CCK‐8 test to evaluate cell viability, b) Western‐blot assay and c) relative protein quantitation of MCP‐1, IL‐6 and Caspase‐3, d) representative images and e) positive percentage of TUNEL stained cells in RPCs, f) representative images and g) fluorescence intensity of ferrous ions using FerroOrange probe, h) representative images and i) relative quantitation of intracellular ROS level upon CM‐H2DCFDA fluorescence probe, j) Western‐blot assay, and k) relative protein quantitation of Rhodopsin, *β*3‐tubulin, and PKC‐*α* were performed. Scale bars: 50 µm in (d), 100 µm in (f) and (h). *n* = 6 in (d) and (e), *n* = 3 in (a–c) and (f–k) one‐way ANOVA with Bonferroni correction, **p < 0.01, not significant (N.S.) > 0.05, data presented as mean ± SD.

Oxidative stress is one of the predominant factors causing progressive dysfunction and death of photoreceptors in RD.^[^ ^26^ ^]^ Because transplanted donor cells encounter excessive oxidative stress in the degenerative retina, we simulated a similar pathological environment using NaIO_3_, which is a typical precursor to retinal‐damaging free radicals, to evaluate the protective antioxidative ability of Nb_2_C MXene on RPCs. Compared to the PBS‐treated control group, a pathological dose of 10 × 10^−3^ m NaIO_3_ induced a greater death rate in RPCs after culture for 24 h when analyzed using CCK8 (*p* < 0.001). A distinct increase in cell viability was observed in NaIO_3_‐induced RPCs treated with Nb_2_C MXene (*p* < 0.001) (**Figure** **4**a), consistent with the results of cell dead/live staining (Figure S9a,b, Supporting Information). An overload of ferrous ions, ROS and lipid ROS (LOS), produced by NaIO_3_ action, comprise a large proportion of damaging free radicals, which are closely associated with inflammatory responses and cell apoptosis.^[^ ^27^ ^]^ NaIO_3_ treatment dramatically upregulated the protein expression levels of the MCP‐1 (*p* < 0.001) and IL‐6 (*p* < 0.001) inflammatory biomarkers, as well as the Caspase‐3 (*p* < 0.001) apoptosis marker in RPCs; these changes were significantly prevented by Nb_2_C treatment (Figure 4b,c). In addition, Nb_2_C mediation in NaIO_3_‐induced cells resulted in a clearly reduced percentage of terminal deoxynucleotidyl transferase dUTP nick end labeling (TUNEL)‐positive cells in comparison to the NaIO_3_ group (11.58 ± 2.93% vs 51.67 ± 5.45%, *p* < 0.001) (Figure 4d,e). These observations provided preliminary evidence that Nb_2_C MXene protects RPCs from NaIO_3_‐mediated oxidative damage. In contrast to the PBS group, RPCs showed a significant accumulation of intracellular ferrous ions when exposed to NaIO_3_, as determined by an increased fluorescence. Nb_2_C treatment remarkably repressed the ferrous ion accumulation that occurred in the presence of NaIO_3_ (*p* < 0.001) (Figure 4f,g). Simultaneously, exposure to NaIO_3_ resulted in an obvious increase in total ROS and LOS levels in RPCs using general oxidative stress indicators such as 2′,7′‐dichlorodihydrofluorescein diacetate (CM‐H2DCFDA) and C11‐4,4‐difluoro‐4‐bora‐3a,4a‐diaza‐s‐indacene (C11‐BODIPY), respectively. NaIO_3_‐treated RPCs exhibited stronger dichlorofluorescein (DCF) and oxidative‐BODIPY fluorescence intensity compared to that shown by untreated RPCs (*p* < 0.001), which was efficiently suppressed by Nb_2_C MXene (*p* < 0.001) (Figure 4h,i; Figure S10a,b, Supporting Information). These results indicated that the protective capability of the Nb_2_C MXene mainly contributes to their effective free radical scavenging activity. Additionally, when in vitro isolated RPCs were treated with Nb_2_C MXene, the downregulation of Rhodopsin, *β*3‐tubulin, and PKC‐*α* proteins in NaIO_3_‐induced RPCs was reversed, which may exclude the effects of the mice retina or the differentiation of grafted RPCs (Figure 4j,k). Taken together, these data demonstrated that Nb_2_C MXene could synergistically serve as potent antioxidants during the mediation of RPC neuronal differentiation.

### 2.5 Effects of Nb_2_C MXene on RPC Differentiation

**Figure 5:**
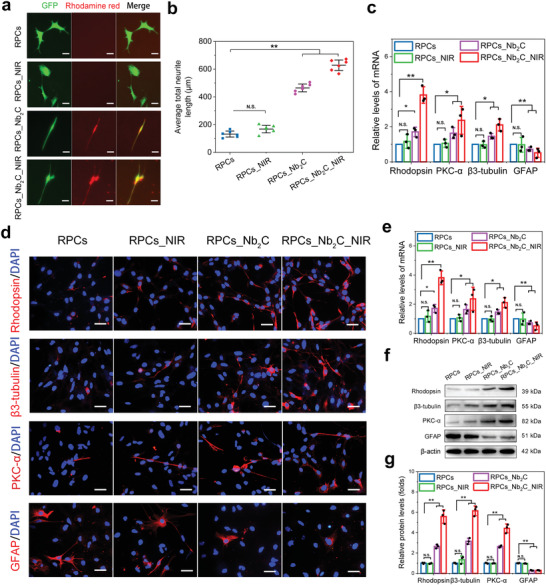
Effects of Nb_2_C MXene on RPC Differentiation. After treatment with NIR, Nb_2_C, or Nb_2_C_NIR for 7 days under differentiation condition, a) divergent morphologies of differentiated RPCs, and b) quantitative analysis of average neurite length were evaluated. Scale bars: 100 µm. *n* = 6, one‐way ANOVA with Bonferroni correction, **p < 0.01, not significant (N.S.) > 0.05, data presented as mean ± SD. c) qPCR analysis of mRNA expression levels of Rhodopsin, *β*3‐tubulin, PKC‐*α* and GFAP in RPCs with NIR, Nb_2_C and Nb_2_C_NIR treatment. *n* = 3, one‐way ANOVA with Bonferroni correction, *p < 0.05, **p < 0.01, not significant (N.S.) > 0.05, data presented as mean ± SD. Protein expression levels of Rhodopsin, *β*3‐tubulin, PKC‐*α* and GFAP were further determined by d,e) immunocytochemical staining and f,g) Western‐blot experiments together with their respective quantification. Scale bars: 50 µm, *n* = 3, one‐way ANOVA with Bonferroni correction, **p < 0.01, not significant (N.S.) > 0.05, data presented as mean ± SD.

During differentiation culture for 7 days, the effects of Nb_2_C MXene with or without NIR exposure once‐daily on the fate of RPCs were investigated by determining divergent cell morphologies, along with the mRNA expression and protein levels of retinal biomarkers. Interestingly, a majority of RPCs incubated with the rhodamine red‐marked Nb_2_C MXene (RPCs_Nb_2_C) displayed dramatically longer neurite structures than that in untreated RPCs, by analyzing neurite length (465.17 ± 28.09 µm vs 132.17 ± 21.78 µm, *p* < 0.001). The neurite length was further enhanced in Nb_2_C MXene upon NIR irradiation (RPCs_Nb_2_C_NIR) (628.5 ± 37.86 µm vs 132.17 ± 21.78 µm, *p* < 0.001) (**Figure** **5**a,b), indicating the promotion of neuron‐like cell differentiation of RPCs using moderate photothermal stimulation. In contrast to the control RPCs, Nb_2_C‐loaded RPCs exhibited markedly higher mRNA expression of retinal neuron biomarkers, including Rhodopsin (a photoreceptor marker, *p* < 0.05), protein kinase C‐alpha (PKC‐*α*, a bipolar cell marker, *p* < 0.05), and *β*3‐tubulin (a neuronal marker, *p* < 0.05), and lower mRNA expression of the retinal glial marker glial fibrillary acidic protein (GFAP, *p* < 0.001) (Figure 5c). More robust changes in these gene expression profiles were identified in the RPCs_Nb_2_C_NIR group, whereas NIR irradiation alone in RPCs produced no obvious difference (*p* = 0.508, *p* = 0.684, *p* = 0.824, and *p* = 0.914, respectively) (Figure 5c). These results suggested that both Nb_2_C MXene and Nb_2_C_NIR promote retinal neuron‐like cell differentiation and simultaneously inhibit astrocyte‐like differentiation of RPCs. In addition, immunocytochemistry analysis revealed that compared to control RPCs, cells stained for Rhodopsin, PKC‐*α*, or *β*3‐tubulin were detected notably more abundant in the RPCs_Nb_2_C and RPCs_Nb_2_C_NIR groups, whereas their prevalence were almost unchanged in NIR‐alone‐irradiated RPCs (Figure 5d,e). As indicated by the respective Western blot, similarly promoted neuronal profiles were observed in RPCs cultured with Nb_2_C, which were further augmented upon NIR irradiation (Figure 5f,g). Overall, Nb_2_C MXene possessed the advantage of substantially accelerating RPCs commitment toward the retinal neuronal network, including photoreceptors. This, with the aiding of mild photothermal therapy upon NIR irradiation, may allow the optimal neuron‐like cell differentiation. It has been reported that carbide‐based biomaterials could induce robust neuronal differentiation,^[^ ^28^ ^]^ which may be another potential mechanism for the improved neuronal differentiation in RPCs upon Nb_2_C administration. Importantly, taking advantage of the photothermal effect in Nb_2_C MXene could pave way for a feasible alternative to regulate RPC differentiation toward retinal neurons and, in turn, enhance RPC‐based therapeutic efficacy for retinal regeneration.

In addition to multidirectional differentiation, RPCs were also able to self‐renew and proliferate, as evaluated by real‐time quantitative polymerase chain reaction (qPCR), immunocytochemistry, and Western blot analyses after Nb_2_C_NIR treatment. After incubation for 3 days under proliferative conditions, the Edu‐stained cell ratio of the RPCs_Nb_2_C_NIR group was similar to that of the control group (55.13% and 55.64%, respectively) (Figure S11a,b, Supporting Information). In comparison to the control group, the mRNA level of Ki‐67 (a cell proliferation marker) was almost unchanged in the RPCs_Nb_2_C_NIR group (Figure S11c, Supporting Information). Western blot analysis revealed that Nb_2_C_NIR‐treated RPCs showed no obvious changes in the protein expression levels of retinal precursor cell biomarkers, including Nestin, Vimentin, and Pax‐6 (Figure S12a,b, Supporting Information). These results indicated that RPCs retain their proliferation and specific stemness profiles after the administration of Nb_2_C_NIR. However, cell migration in the transplanted areas is essential for retinal repair. The horizontal and vertical migration of RPCs were promoted when Nb_2_C_NIR was administered for 72 h in wound healing and Transwell assays, respectively (Figure S13a–c, Supporting Information). Improved horizontal cell migration is beneficial for the scope of transplantation, whereas vertical migration supports the architecture recovery of host retina after cell transplantation. The collective data provided preliminary confirmation that Nb_2_C with NIR irradiation strengthen the efficiency of Nb_2_C MXene in promoting the neuronal differentiation of RPCs. However, the initial molecular changes and mechanisms associated with the augmented neuronal differentiation require further investigation.

### 2.6 Mechanism Underlying Nb_2_C_NIR‐Mediated RPC Neuronal Differentiation

**Figure 6:**
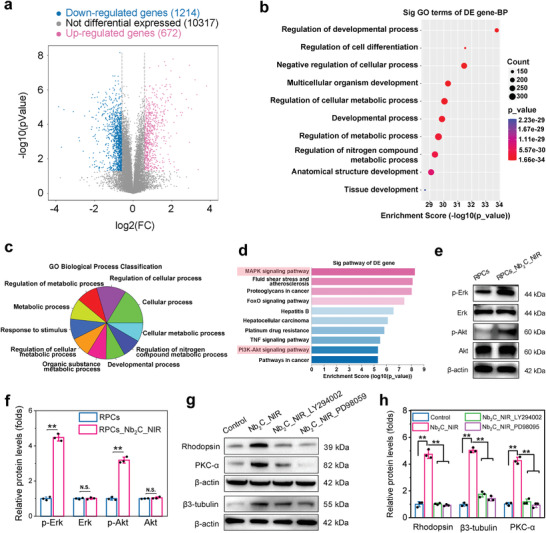
Mechanism underlying Nb_2_C_NIR‐mediated RPC neuronal differentiation. a) Volcano plot by RNA sequencing displayed differentially expressed genes of Nb_2_C_NIR‐treated RPCs against untreated RPCs. *p* < 0.05, |fold change| ≥ 1.5. b) Top ten of GO terms were obtained by gene enrichment analysis of differentially upregulated genes after Nb_2_C_NIR treatment. c) A pie chart showed GO biological process classification among differentially upregulated genes. d) KEGG analysis enriched the top ten signaling pathways among the differentially upregulated genes. e) Western blot analysis of Akt and ERK phosphorylation. f) Quantitative evaluation of the relative expression levels of p‐Akt and p‐ERK. *n* = 3, two‐tailed Student's t‐test, **p < 0.01, not significant (N.S.) > 0.05, data presented as mean ± SD. g) Western blot analysis of the protein expression levels of Rhodopsin, *β*3‐tubulin, and PKC‐*α* in Nb_2_C_NIR‐treated RPCs in the presence of Akt inhibitor (LY294002) and Erk inhibitor (PD98059). h) Quantitative evaluation of the relative expression levels of Rhodopsin, *β*3‐tubulin, and PKC‐*α*. *n* = 3, one‐way ANOVA with Bonferroni correction, **p < 0.01, data presented as mean ± SD.

High‐throughput RNA sequencing was performed to understand the inherent molecular mechanisms involved in the enhanced neuronal differentiation in RPCs_Nb_2_C_NIR. Compared with untreated RPCs, RPCs_Nb_2_C_NIR displayed a significant change in the expressed gene program. Volcano plot and heat map analyses identified 672 differentially upregulated and 1214 downregulated genes (**Figure** **6**a; Figure S14a, Supporting Information). To identify whether cellular processes, functions, and components were affected by Nb_2_C_NIR exposure, gene ontology (GO) enrichment techniques were applied. Focusing on the top 10 GO terms enriched by differentially upregulated genes involved in cell biological processes, the crucial terms retrieved were specific to regulation of developmental process, regulation of cell differentiation, developmental process, and tissue development (Figure 6b). Especially, the enrichment score of “Regulation of differentiation” is 31.53 and ranked at the second among the top 10 terms, although the size of the dot (representing the number of genes enriched into this terms) in “Regulation of differentiation” is the smallest. This enrichment highlighted that a more selective group for cell differentiation was influenced by the physical and chemical properties of the Nb_2_C MXene. In addition, the top 10 GO terms enriched by differentially downregulated genes were also associated with the cell cycle, mitotic cell cycle, regulation of cell cycle, and the regulation of cell cycle process, clearly indicating cell cycle exit (Figure S14b, Supporting Information). The progression of development from the embryo to tissue or organ is associated with the coordination of differentiation and proliferation in progenitor/stem cells.^[^ ^29^ ^]^ Accordingly, driving the cells to exit the cell cycle upon Nb_2_C‐NIR treatment makes it reasonable to further explore the mechanism behind improved retinal neuronal differentiation in RPCs. We, likewise, analyzed the GO biological process clusters from differentially upregulated genes. Moreover, clear distinction emerged pertaining to responses to stimuli, developmental processes, cellular processes, and regulation of cellular processes (Figure 6c). We suspected that Nb_2_C MXene attaches to the cell membrane and subsequently internalizes into cells initiates the cellular response to stimuli, thereby enabling direct RPC‐specific differentiation toward neural lineages.

To further provide transcriptomic insight into 2D MXene‐mediated cellular pathways, clustered signaling pathways of differentially upregulated genes were identified using the Kyoto Encyclopedia of Genes and Genomes (KEGG). Most notably, marked activation of mitogen‐activated protein kinase (MAPK) and phosphatidylinositol 3‐kinase/protein kinase B (PI3K)‐Akt pathways was observed in the RPCs_Nb_2_C_NIR group (Figure 6d). The MAPK signaling pathway is the most critical among the pathways mediated by biological and chemical cues, and can improve neurite outgrowth and direct specific neuronal differentiation.^[^ ^30^ ^]^ Similarly, activation of the PI3K‐Akt pathway reportedly regulates cell differentiation and provides neuroprotection in neurons.^[^ ^31^ ^]^ Importantly, mild photothermal stimulation is also presumed to positively guide cell differentiation through the extracellular signal‐regulated kinase (ERK)1/2 and PI3K‐Akt pathways.^[^ ^32^ ^]^

To evaluate whether MAPK and Akt signaling pathways were activated, we assessed the phosphorylation of Erk (p‐Erk) and Akt (p‐Akt) in RPCs following Nb_2_C‐NIR exposure. Compared to control RPCs, a 3.2‐fold and 2.7‐fold increase in protein expression levels of p‐Erk (*p* < 0.001) and p‐Akt (*p* < 0.001), respectively, were observed in RPCs_Nb_2_C_NIR (Figure 6e,f), indicating the activation of MAPK and PI3K‐Akt pathways in Nb_2_C_NIR‐induced RPC differentiation. Additionally, when RPCs were precultured with LY294002 (an Akt antagonist) and PD98059 (an Erk inhibitor), the upregulated protein levels of Rhodopsin, *β*3‐tubulin, and PKC‐*α* in Nb_2_C_NIR‐treated RPCs were suppressed (Figure 6g,h). These observations suggested that Nb_2_C_NIR‐mediated RPC neuronal differentiation is relevant to the activation of both MAPK and PI3K‐Akt signal transduction pathways, which could be helpful to better understand the nanomaterial–cell interactions.

Oxidative stress can impair mitochondrial function and result in cell death.^[^ ^33^ ^]^ We have shown that oxidative stress induced RPCs damage in Figure 4, which could be ameliorated by Nb_2_C. As presented in Figure 3g, the Nb_2_C MXene mainly distributed in the mitochondrion of RPCs, implying that Nb_2_C alleviated oxidative damage possibly by regulating oxidative stress‐mediated mitochondrion malfunction. In addition, it has been reported that oxidative stress‐induced mitochondrial dysfunction involves two important survival signaling mechanisms in cells, i.e., the PI3K‐Akt and MAPK pathways, and upregulation of the two signaling pathways exerts a cytoprotective effect against oxidative stress‐mediated mitochondrial impairment.^[^ ^34^ ^]^ Likewise, in this work, Nb_2_C_NIR significantly activated MAPK and PI3K‐Akt signal transduction pathways upon RNA‐Seq and biomedical experiments, implicating that Nb_2_C_NIR may defense against oxidative stress‐induced mitochondrial injury via activating MAPK and PI3K‐Akt pathways during the mediation of RPC neuronal differentiation, though these required further evaluation.

### 2.7 RPCs_Nb_2_C_NIR Transplantation Rescued Retinal Structure and Function of rd10 Phenotype

Retinal degeneration 10 (rd10) mouse model bears a missense mutation (R560C) of the Pde6*β* gene that leads to photoreceptor dysfunction and degeneration. Usually, rd10 mouse displays a relatively slower photoreceptor degeneration starting from postnatal day 17 (P17) and until P60 when most of the photoreceptors disappear.^[^ ^35^ ^]^ Furthermore, given a relatively later onset and slower retinal degeneration that could mimic more closely the human conditions, rd10 mouse is an eligible model to understand the pathogenesis and study the therapeutic possibilities for retinal restoration. Thus, we investigated the effects of Nb_2_C_NIR‐treated RPCs (RPCs_Nb_2_C_NIR) on the photoreceptor phenotype and visual function in a rd10 mouse model over the long term, and the age‐matched wild‐type (WT) C57/BL6J served as the blank control group.

**Figure 7:**
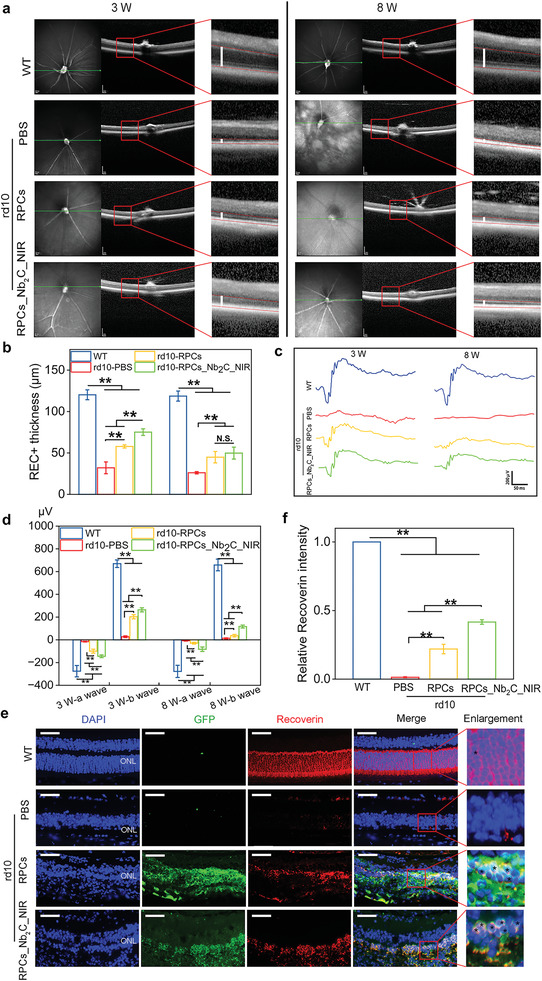
Retinal structural and functional rescue of the rd10 phenotype upon RPCs_Nb_2_C_NIR transplantation. After transplantation of RPCs alone or Nb_2_C_NIR‐treated RPCs for 3 weeks (at P35) and 8 weeks (at P70), the age‐matched healthy WT mice were used as blank control, a) representative SD‐OCT photos were imaged, and b) statistically analyzed the thickness of REC+ (photoreceptor + RPE) obtained from the segmentation of SD‐OCT images. SD‐OCT: spectral domain optical coherence tomography. Scale bars: 200 µm. *n* = 6, one‐way ANOVA with Bonferroni correction, **p < 0.01, not significant (N.S.) > 0.05, data presented as mean ± SD. c) Electroretinography recording and d) statistical analysis of the a‐wave and b‐wave in the P35 and P70 mouse retina harvested from either WT or rd10 mice transplanted with PBS, RPCs or RPCs_Nb_2_C_NIR. *n* = 6, one‐way ANOVA with Bonferroni correction, **p < 0.01, data presented as mean ± SD. e) Representative immunostaining images and f) statistical analysis of Recoverin‐positive cells (red) (black stars in the enlarged pictures) from either WT or rd10 mice retinas 8 weeks after transplantation of PBS, GFP^+^ RPCs or GFP^+^ RPCs_Nb_2_C_NIR. ONL: outer nuclear layer. Scale bars: 50 µm. *n* = 6, one‐way ANOVA with Bonferroni correction, **p < 0.01, data presented as mean ± SD.

Subretinal injection of RPCs or RPCs_Nb_2_C_NIR into rd10 mouse retina was performed at P14 when photoreceptors were relatively normal. Following treatment for 3 weeks (at P35), a significant thinning of the REC+ (photoreceptor + RPE) thickness across the segmentation of SD‐OCT images from rd10 mice treated with PBS was observed in contrast to the WT group (32.05 ± 7.16 µm vs120.27 ± 6.0 µm, *p* < 0.001), confirming the almost complete loss of photoreceptors in 35‐day‐old rd10 mice. It is noteworthy that the REC+ thickness in retinas from RPCs‐engrafted P35 rd10 mice increased (57.91 ± 1.95 µm vs 32.05 ± 7.16 µm, *p* < 0.001), which was more significantly in rd10 mice treated with Nb_2_C_NIR‐treated RPCs (75.2 ± 4.01 µm vs 32.05 ± 7.16 µm, *p* < 0.001) (**Figure** **7**a,b). The data primarily suggested that photoreceptors in rd10 mouse retina with cell transplantation was partly rescued, and RPCs_Nb_2_C_NIR transplantation showed a better improvement than RPCs alone. To observe the long‐term effects of cell transplantation on rd10 mouse, we further performed the SD‐OCT after administration for 8 weeks (at P70). In comparison to the PBS‐treated rd10 mouse (26.07 ± 1.4 µm), the REC thickness in rd10 mouse with RPCs and RPCs_Nb_2_C_NIR implantation was still maintained at 44.9 ± 6.87 µm (*p* < 0.001) and 49.84 ± 7.22 µm (*p* < 0.001), respectively, which indicated the sustained protection of cell transplantation.

Electrophysiological response is an important clinical indicator of visual function and is closely correlated with the remaining functional photoreceptors.^[^ ^7a^ ^]^ To evaluate whether grafted cells are able to rescue the visual function of rd10 phenotype, we recorded scotopic electroretinographic (ERG) responses of rd10 mouse after dark adaption. As presented in Figure 7c,d, the ERG amplitudes recorded from 35‐day‐old rd10 mouse treated with PBS became sluggish (−15.57 ± 3.38 µV in a‐wave mean; 27.32 ± 7.33 µV in b‐wave mean). Instead, rd10 retinas transplanted with RPCs for 3 weeks exhibited an increase in ERG a‐waves and b‐waves (−99.4 ± 17.32 µV in a‐wave mean; 202.88 ± 18.31 µV in b‐wave mean), and a more significant improvement was achieved in the RPCs_Nb_2_C_NIR treated rd10 mouse (−144.5 ± 11.57 µV in a‐wave mean; 263.62 ± 18.02 µV in b‐wave mean), despite these were lower than the WT levels (−275.83 ± 50.14 µV in a‐wave mean; 669.28 ± 33.32 µV in b‐wave mean). However, the ERG responses became flat in rd10 mice following transplantation of RPCs for 8 weeks. Though, statistically, Nb_2_C_NIR_RPCs treatment still showed a more significantly recordable amplitude in the a‐wave (−82.7 ± 19.46) and b‐wave (116.69 ± 13.17 µV) than that of RPCs transplantation (−29.2± 7.8 µV in a‐wave mean; 37.16 ± 11.21 µV in b‐wave mean) upon strong flash intensities (10 cd s m^−2^). These findings implied the successful production of functional photoreceptors from engrafted Nb_2_C_NIR_RPCs within the host's retina to delay the overall retinal degeneration.

At the histological level, we determined on retinal sections whether RPCs and Nb_2_C_NIR‐treated RPCs are able to produce photoreceptors in a long‐term period. Through IF staining of mature photoreceptor marker (Recoverin), we found that the ONL cells were clearly stained with Recoverin in age‐matched WT C57/BL6J mice. In contrast, at week 8 after transplantation, Recoverin staining was drastically decreased and even undetectable in retinas from PBS‐treated P70 rd10 mice, ascribing to the complete photoreceptor degeneration. Instead, ≈2–3 rows of cell nuclei in the rebuilt outer nuclear layer (ONL) were observed upon GFP^+^ RPCs transplantation, and some of these were positive for Recoverin expression (Figure 7e,f). Remarkably, after transplantation of Nb_2_C_NIR‐treated GFP^+^ RPCs, we found a majority of them were co‐stained with Recoverin. These results strongly indicated that transplanted RPCs could commit toward photoreceptors in the host degenerated retina, which was further enhanced by Nb_2_C_NIR‐treated RPCs, validating the potential of biomaterials‐aided xeno cells transplanted into the degenerative host retina for architecture and visual restoration.

Gliosis is a pathological change occurring in retinal degeneration, which plays an essential role in determining the fate of transplanted cells.^[^ ^36^ ^]^ Reactive gliosis in retina is characterized by the upregulated expression of glial fibrillary acidic protein (GFAP) in activated Müller cells, and rd10 mouse model is accompanied by gliosis activation. As shown in Figure 5, we have demonstrated that Nb_2_C_NIR enhanced the neurogensis of RPCs while inhibited the gliosis. To further investigate the antigliosis effect of Nb_2_C_NIR in vivo, we evaluated the GFAP expression in the WT and rd10 retina by immunostaining. As presented in Figure S15 (Supporting Information), in normal retina of WT mice, minimal levels of GFAP were mainly expressed in astrocytes located in the ganglion cell layer (GCL), and the GFAP staining extraordinarily extended along the processes of activated Müller cells across the whole retinal layers in the P70 rd10 mice with PBS treatment. Compared to the PBS‐treated rd10 retina, RPCs alone transplantation in rd10 retina exhibited a similar upregulation but shorter average length of GFAP‐positive immunolabelling, while a significantly decreased immunoreactivity of GFAP in Müller cells in rd10 mice grafted with Nb_2_C_NIR‐treated RPCs was observed, suggesting a suppression of gliosis by Nb_2_C_NIR in the degenerative retina.

Cell fusion and material transfer have been considered as potential mechanism of cell‐based regenerative therapy in the retinal degeneration. Cell fusion by combination of stem cell and host cell and their nuclei has been identified in some cases.^[^ ^37^ ^]^ Sanges et al. transplanted hematopoietic stem and progenitor cells into degenerated retinas and found spontaneous cell fusion between grafted cells and host Müller glia. The Müller‐donor cell hybrids effectively differentiated into functional photoreceptors in a mouse model of retinitis pigmentosa.^[^ ^37b^ ^]^ Fusion of transplanted cells may deliver key factors to the targeted cells, but the process of cell nuclear fusion is relatively slow.^[^ ^38^ ^]^ Presently, some studies have described that material transfer appears to account for a significant proportion of transplanted cells within the recipient retina.^[^ ^39^ ^]^ The exchange of functionally important DNA and/or proteins of photoreceptors likely explains the vision rescue in blind mice with photoreceptor transplantation.^[^ ^40^ ^]^ However, the material transfer between donor and host cells usually requires the remaining host photoreceptors, which may yet to be proven optimal for cell support therapy under different conditions.^[^ ^41^ ^]^ Additionally, the historical interpretation of visual functional rescue has centered around the migration and integration of donor cells into recipient retina, and these cells maturated into functional photoreceptors for host‐graft synaptic reconstruction.^[^ ^42^ ^]^ Recently, Gasparini et al. observed that, during incorporation and maturation of transplant, the activated Müller glia intermingled throughout the graft.^[^ ^43^ ^]^ Rather than forming a glial barrier, the Müller glia participated in establishing a series of adherent junctions between donor and host cells, while whether the activated Müller glia directly improved graft maturation remains to be proven. Transplantation outcomes are largely dependent upon the developmental stage of the donor cell and host environment, and the specific mechanisms underlying host‐graft visual network reconstruction remain to be determined and will require a large number of experimental evidences.

In current study, the GFP^+^ cells originated from the RPCs and Nb_2_C_NIR_RPCs were tracked over the long term, i.e., 8 weeks after transplantation in rd10 mice. Compared with RPCs, Nb_2_C_NIR‐treated RPCs demonstrated a more efficient differentiation into photoreceptors in rd10 mice, and a more significantly visual protection of the regenerated photoreceptors upon to remodel ONL. The Pde6*β* gene mutation in rd10 mouse was associated with oxidative stress and ER stress, leading to the gradual photoreceptor degeneration.^[^ ^44^ ^]^ Thus, either regenerative or protective effects for the ONL neurons after RPCs_Nb_2_C_NIR treatment may ascribe to the unique antioxidative stress of Nb_2_C Mxene and Nb_2_C Mxene‐induced efficient photoreceptor differentiation.

## 3 Conclusion

In summary, the photothermal‐conversional and nanotherapeutic Nb_2_C MXene effectively improved retinal neuronal differentiation of RPCs and synergistically protected RPCs against undesirable oxidative damage by moderate photothermal effect and free radical scavenging, respectively. Importantly, Nb_2_C MXene was degradable without in vivo metabolism problem, and possessed highly biocompatible as confirmed by systematic biosafety evaluations. By employing the model of retinitis pigmentosa, MXene‐aided treatment was shown to notably promote the neuronal differentiation of transplanted RPCs in the host retina, thereby contributing to the rescue of retinal construction and the preservation of visual function.

In our future work, further investigations in larger animals would be considered to make the conclusion more convincible. Moreover, the 8 weeks of observation period after transplantation is relatively short. It is necessary to prolong the postoperative follow‐up period and track transplanted cell integration. Additionally, the time of intervention is essential for photoreceptor functional regeneration and protection. Cell transplantation at the late stage of retinal degeneration, when a complete degeneration of photoreceptor occurred, theoretically, is difficult to revert the cells in the ONL. It is worth considering that the photoreceptor degeneration progress is much slower in human pathology than that in the rd10 mice, suggesting that the intervention time window will be considerably larger and the optimal intervention time point for cell transplantation necessaries prudent consideration. Although further intricacies need to be elucidated, the fundamental biological significance and clinical relevance demonstrated in the present work indicates the ultrathin Nb_2_C MXene to be potential in assisting progenitor/stem cell transplantation for treating patients with retinal degeneration and related diseases by synergistically serving as a powerful antioxidant and driver of neuronal differentiation.

## 4 Experimental Section

### Chemicals and Reagents

DMEM/F12 medium, penicillin/streptomycin, CM‐H2DCFDA fluorescence probe, Tunel staining reagent, live/dead reagent and fetal bovine serum (FBS) were obtained from Sigma‐Aldrich. NaIO_3_, Erk inhibitor (PD98059) and Akt inhibitor (LY294002) were purchased from selleck chemicals (Houston, TX, USA). FerroOranege probe, cell proliferation reagent of cell light 5‐ethynyl‐20‐deoxyuridine (Edu) and cell counting kit‐8 (CCK‐8) kit were obtained from Guangzhou RiboBio, China, and Dojindo (China Co., Ltd, China). Other chemicals were brought from Sigma‐Aldrich unless mentioned otherwise

### Synthesis of Nb_2_C MXene

10 g of Nb_2_AlC ceramic powder was added into HF aqueous solution (50 wt%, 60 mL), the mixture solution stirred at room temperature for 48 h, followed by centrifugation for 10 min. The obtained precipitate was washed with ethanol (EtOH) and H_2_O for twice, respectively, dispersed in tetrapropylammonium hydroxide (TPAOH, 25 wt%, 60 mL), and stirred at room temperature for 72 h. After centrifugation for 10 min, the precipitate was washed three times with EtOH and H_2_O to obtain Nb_2_C MXene.

### Surface Engineering of Nb_2_C MXene

Nb_2_C MXene and polyvinylpyrrolidone (PVP, average MW 40 000) were dispersed in 100 mL of EtOH and heated at 50 °C for 12 h. Then, the mixture solution was centrifuged and washed with EtOH and H_2_O for three times, PVP modified Nb_2_C MXene was dispersed in water for further use.

### In Vitro Photothermal Performance of Nb_2_C MXene

Various concentrations of Nb_2_C MXene were irradiated by 808 or 1064 nm lasers at power density of 1.0 W cm^−2^ for 10 min. Additionally, Nb_2_C aqueous solution (50 µg mL^−1^) was exposed to 808 or 1064 nm lasers at different power densities. An infrared (IR) thermal imaging camera was used to record temperature changes and thermal images of Nb_2_C MXene under 808 or 1064 nm lasers exposure.

### Computational Section

Geometry optimization and energy calculations of the Nb_2_C systems were performed by using density functional theory (DFT) method implemented VASP.^[^ ^45^ ^]^ The exchange‐correlation function was generalized gradient approximation (GGA) of Perdew–Burke–Ernzerhof (PBE).^[^ ^46^ ^]^ The projector‐augmented wave (PAW) method was used to treat the interaction between the ionic cores and valence electrons. The electronic plane wave interception energy was set to be 350 eV and a 4 × 4 × 2 *k*‐point Gamma mesh is used for the models of orthogonal supercell (3 × 3 × 1‐unit cells of Nb_2_C unit cell) with periodic boundary conditions (PBC) containing 9 Nb_2_C pairs to ensure adequate convergence. The vacuum layer between neighboring images were set to be larger than 15 Å, which is enough to avoid the interactions between neighboring images. All these structures were relaxed until the energy differences were converged within 10^−4^ eV and the forces of all atoms were less than 0.01 eV Å^−1^. The semiempirical DFT‐D3 correction method was employed to describe the van der Waals (vdW) interactions between Nb_2_C and other molecules.^[^ ^47^ ^]^

### Isolation and Culture of RPCs

In this study, RPCs were associated from GFP^+^ C57BL/6 mice at postnatal day 1.^[^ ^48^ ^]^ Isolated fresh retinal tissues were digested and then cultured with proliferation medium containing 1% N_2_ neural supplement, 2 × 10^−3^ m l‐glutamine, advanced DMEM/F12, and 20  ng mL^−1^ epidermal growth factor (EGF). Differentiation medium consisting 10% FBS without EGF were used for RPC differentiation culture. Isolated GFP^+^ cells were characterized by detecting the expression levels of neural progenitor‐related markers, including Nestin, Vimentin and Pax‐6, together with the glial cell marker GFAP through immunocytochemistry (as described below).^[^ ^22^ ^]^ The neurite length of differentiated RPCs was imaged using microscopy (Nikon) and then calculated by ImageJ software (NIH, MD).

### Internalization of MXene by RPCs

RPCs cultured with or without Nb_2_C MXene (50 µg mL^−1^) for 24 h were collected for evaluation of encapsulated MXene in RPCs by transmission electron microscopy (TEM). Collected RPCs were prefixed in 2.5% glutaraldehyde phosphate (pH = 7.4, Science Services) for 8 h at 4 °C, then postfixed in buffered osmium tetraoxide (2%) and embedded within Epon812 (Merck). The 60 nm thick of ultrathin sample sections was cut, and stained with uranyl acetate and lead citrate. TEM microscopy (FEI, Hillsboro, OR, USA) was used to take pictures.

### Cell Viability Analysis

Live/dead reagent was applied to stain the dead and live RPCs (4 × 10^4^ cells well^−1^, 24‐well plates) as the previous study reported.^[^ ^49^ ^]^ Treated RPCs were incubated with live/dead kit for 30 min. Following washing with PBS, stained RPCs were imaged by fluorescence microscope (Nikon), and ImageJ software was used to count the live and dead RPCs. Cell viability was presented as (live cells/total cells in the field) × 100%.

In addition, RPCs were plated in 96‐well plates (1 × 10^4^ cells per well) using CCK‐8 reagent to evaluate proliferation capability and cell viability according to manufacturer's instructions. Treated RPCs were supplemented with CCK‐8 reagent (10 µL well^−1^) at determined time for 2 h. The optical density at 450 nm (values of O.D. 450 nm) was detected under ELISA microplate reader (ELX800, BioTeK, USA). Final cell viability was calculated as fold changes relative to control by measuring the values of O.D. 450 nm.

### Migration Analysis

Transwell and scratching assays were performed to investigate cell vertical migration and horizontal migration, respectively. Briefly, for transwell assay, RPCs were plated on cell culture inserts (8.0 µm pores, 24‐well plates) and culture medium were added into the upper and lower chambers. Following culture for 24 h, cells in inserts were fixed with 4% paraformaldehyde for 30 min, and nonmigrated RPCs were wiped off from the upper surface of the inserts. 1% Crystal Violet was used to stain vertically migrated RPCs for 15 min and washed with PBS. Migrated RPCs were imaged upon microscope and calculated by ImageJ software.

For scratching assays, 200 µL pipet tip was utilized to scratch RPCs after that RPCs grown at 90% confluence to cause a “wound area.” PBS was used to wash the scraped cell debris. The photographs of wound healing area were imaged at 0, 36, and 72 h after scratch, and the lateral cell migration ability was evaluated and quantified using ImageJ software.

### Proliferation Analysis

Edu kit was used to investigate the proliferating RPCs according to manufacturer's instructions. Edu medium mixture was added to the treated RPCs. Following incubation for 30 min, 4% paraformaldehyde was applied to fix RPCs for 30 min at 37 °C and stained with Apollo Dye Solution. Hoechst 33342 was used to stain nucleic acids for 10 min. Fluorescent microscope was performed to image proliferating cells, and RPC proliferation ability was calculated as (Edu‐positive cells/all cells in the field) ×100%.

### qPCR Analysis

RPCs plated on 6 cm dishes (3 × 10^5^ cell dish^−1^) were cultured under differentiation condition for 7 days or proliferation condition for 3 days. Total RNAs in RPCs were collected by TRIzol reagent as recommended procedures. The qPCR was performed using Real‐Time PCR Detection System (Applied Biosystems) to measure gene expressions of MCP‐1, IL‐6, Caspase‐3, Ki‐67, PKC‐*α*, *β*3‐tubulin, Rhodopsin, and GFAP in RPCs. Specifically, their parameters were presented in Table S1 (Supporting Information). Relative mRNA expressions of target genes were normalized to that of an endogenous reference *β*‐actin.

### Immunocytochemical Staining

RPCs seeded on 24‐well plates were cultured under differentiation condition for 7 days. At determined time, treated RPCs were blocked using TBS buffer consisting of 10% normal goat serum (Sigma‐Aldrich) and 0.3% Triton X‐100 after fixing with 4% paraformaldehyde for 30 min at 4 °C. Then, samples were reacted with mouse or rabbit monoclonal antibodies (Table S2, Supporting Information), including anti‐Rhodopsin (mouse), *β*3‐tubulin (rabbit), PKC‐*α* (rabbit), GFAP (mouse), anti‐Vimentin (rabbit), anti‐Pax‐6 (rabbit), and anti‐Nestin (rabbit), for 8 h at 4 °C, and subsequently were counterstained with Alexa Fluor 546 goat antirabbit or mouse secondary antibodies (Sigma‐Aldrich, 1:800). Finally, cell nuclei were incubated with DAPI and representative pictures were visualized using fluorescent microscope. Positive cells were counted by ImageJ software and the positive ratio was presented as (stained cells/all cells in the field) × 100%.

### Western Blot Analysis

Total proteins were collected in prepared RPCs after culture with proliferation medium for 3 days or differentiation medium for 7 day, and then were measured using BCA protein Kit (Pierce, Rockford, IL). Subsequently, SDS‐PAGE (Bio‐red) electrophoresis was utilized to separate proteins, which then were transferred to PVDF membranes (Sigma‐Aldrich), and reacted with primary rabbit or mouse antibodies (Table S2, Supporting Information). The horseradish peroxidase‐conjugated antirabbit or mouse secondary antibodies (Sigma‐Aldrich) were used and the protein blots were imaged using ECL Plus Western Blot Detection Kit (Tanton). Relative protein expression levels were displayed as fold changes in comparison to control.

### RNA Sequencing Analysis

RPCs treated with or without Nb_2_C MXene for 24 h upon irradiation of 1064 nm laser were collected for RNA sequencing and further analysis. Sequenced reads were aligned to the human reference genome (hg19, Genome Reference Consortium GRCh37) utilizing Hisat2 software (version 2.1.0). Gene expression levels were represented as the fragments per kilobase of transcript per million reads (FPKM) based on Ballgown software (version 2.10.0). Absolute value of log_2_ (fold change) ≥1.5 were performed to determine the differentially expressed genes.

### Animal

The retinal degenerations 10 (rd10) mice and wild‐type (WT) C57BL/6J mice were purchased from the Cyagen Biosciences (Suzhou, China) Inc. Mice were housed in standard conditions with laboratory food and water. All procedures were carried out according to the NIH Guide for the Care and Use of Experimental Animals and approved by the institutional animal care and committee of Ninth People's Hospital, Shanghai Jiao Tong University School of Medicine.

### Subretinal Space Injection

Subretinal space injection was performed in accordance with the previously reported method.^[^ ^22^ ^]^ Before operation, mice were anesthetized with isoflurane/oxygen (induction: 3% at 1.0 L min^−1^; maintenance: 1.5% at 0.6 L min^−1^; Suzhou kunchen Biotechnology Co., Ltd),^[^ ^49^ ^]^ and pupils were dilated with 1% tropicamide. A 30‐gauge ½ syringe was utilized to puncture the limbus to decrease intraocular pressure and subsequent cell reflux at the injected site. To evaluate the in vivo degradation behavior of Nb_2_C MXene, subretinal space of mice was injected with 1 µL Nb_2_C Mxene in the right eyes, and with 1 µL PBS in the left eyes as control using a sterile 32‐gauge needle (Hamilton, Reno, NV). For cell transplantation, mice were subretinally injected with 1 µL RPCs or RPCs_Nb_2_C_NIR suspensions (1 × 10^5^ cells) in the right eyes, and with 1 µL PBS in the left eyes as control. At determined time, mice were anesthetized or sacrificed for subsequent experiments.

### In Vivo Degradation Behavior of Nb_2_C Mxene

WT C57BL/6J mice were subretinally administered with Nb_2_C Mxene (1 mg mL^−1^) to investigate the in vivo biodegradation behavior and metabolism process. Main organs (including retina, heart, liver, and kidney), as well as urine and feces sample were collected at different time intervals postinjection (24 h, 1 d, 2 d, 4 d, and 14 d). Dissected tissue samples were then weighed, homogenized, and dissolved using strong acid. Nb element was directly determined by ICP‐AES to quantitative analysis of the biodegradation.^[^ ^50^ ^]^ The biodistributions of Nb_2_C MXene in different organs were calculated as the percentage of injected dose (ID) per gram of tissue (Nb % ID/g).

### Spectral Domain Optical Coherence Tomography (SD‐OCT)

In vivo SD‐OCT scans (Heidelberg Engineering, Heidelberg, Germany) were performed to investigate the retinal phenotype of rd10 mice on weeks 3 and 8 after subretinal transplantation, and age‐matched WT C57/BL6J mice served as blank control. The main landmark was the cross‐sectional images centered on the optic nerve head, and the system was modified according to instruction of the manufacturer to correct mouse optics.^[^ ^51^ ^]^ To detect the thickness of photoreceptors, REC+ (photoreceptor + RPE) from segmentation of SD‐OCT images was determined as the thickness from the base of the RPE to the interface of the inner nuclear layer and outer plexiform layer.

### TUNEL Analysis

According to manufacturer's protocol, TUNEL staining by Cell Death Detection Kit was performed on in vitro cells or retinal sections (8 µm). Samples were fixed in 4% paraformaldehyde and blocked with 0.1% Triton X‐100. Subsequently, they were reacted with the TUNEL mixture consisting of TdT and TMR‐dUTP for 1 h at 37 °C in the dark. After washing, fluorescence microscopy was used to image the label incorporated sites of DNA damage. TUNEL‐stained cells were calculated and the positive ratio was expressed as percent of total cells undergoing cell death.

### Electroretinography (ERG)

Until the 3 and 8 weeks after implantation, ERG was recorded to exam the effects of transplanted cells on visual function. Previous to ERG recordings, all mice were dark‐adapted for 12 h and then anesthetized. Following dilation of pupils by 1% topical tropicamide, each cornea of fixed mice was placed with a gold loop electrode, tail was subcutaneously positioned with a ground lens electrode, and the head region was subcutaneously placed in the reference electrodes. The a‐ and b‐waves in scotopic ERG were elicited at the flash intensities of 10 cd s m^−2^ utilizing a ganzfeld Color Dome stimulator (Espion E3 machine, Diagnosys, MA). All process was performed at the same time of day.

### Retinal Immunofluorescent Analysis

At determined time, eyes of treated mice were immediately collected and embedded in paraffin following fixation to obtain retinal sections. High‐quality retinal sections were stained with targeted primary antibodies for immunofluorescent analysis or directly stained with DAPI for cell viability and migration evaluation. The thickness of outer nuclear layer was calculated in 4 random fields at the temporal and nasal sides of optic nerve head.

### Statistical Analysis

Each result was produced from at least three replicates. Data were presented as the mean ± standard deviation (SD) in corresponding figure legends. Upon the GraphPad Prism 8.0 software for statistical analyses, the unpaired two‐tailed Student's *t*‐test was used for comparison of two groups, and one‐way Variance (ANOVA) with Bonferroni correction was performed to compare multiple groups. *p* < 0.05 was regarded to be statistically significant. *p < 0.05, **p < 0.01, and N.S. indicates no significance.

## Conflict of Interest

The authors declare no conflict of interest.

## Supporting information

Supporting Information

Supplemental Video 1

## Data Availability

The data that support the findings of this study are available from the corresponding author upon reasonable request.
