# Supplementary material for: Bifunctional MXene‐Augmented Retinal Progenitor Cell Transplantation for Retinal Degeneration
Source: Adv Sci (Weinh). 2023 Jun 28;10(25):2302747. doi: 10.1002/advs.202302747 (PMC10477897; doi:10.1002/advs.202302747)
Supplement: Supplementary file 1 — Supporting Information [file ADVS-10-2302747-s001.pdf]

ADVANCED SCIENCE
Open Access

Supporting Information

for *Adv. Sci.*, DOI 10.1002/advs.202302747

Bifunctional MXene-Augmented Retinal Progenitor Cell Transplantation for Retinal Degeneration

Zhimin Tang, Yan Liu, Huijing Xiang, Xinyue Dai, Xiaolin Huang, Yahan Ju, Ni Ni, Rui Huang, Huiqin Gao, Jing Zhang, Xianqun Fan, Yun Su, Yu Chen* and Ping Gu**

Supporting Information

Bifunctional MXene-Augmented Retinal Progenitor Cell Transplantation for Retinal Degeneration

Zhimin Tang, Yan Liu, Huijing Xiang, Xinyue Dai, Xiaolin Huang, Yahan Ju, Ni Ni, Rui Huang, Huiqin Gao, Jing Zhang, Xianqun Fan, Yun Su, Yu Chen*, Ping Gu**

Supporting Information includes:

S1. Supporting Figures S1 ~ S15

S2. Supporting Tables S1 ~ S2

S1. Supporting Figures S1~S15.

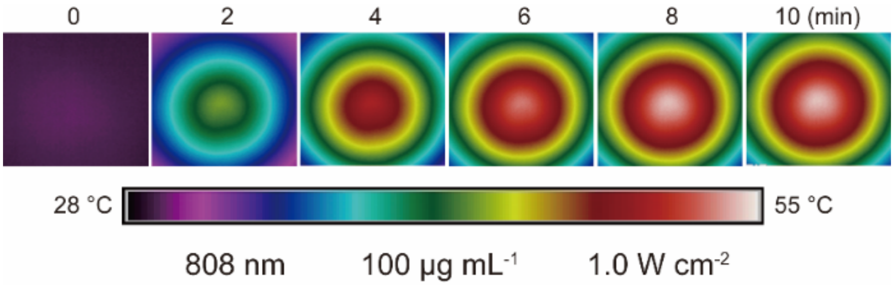

Figure S1. Thermal images of Nb_2C MXene (100 μg mL^{-1}) under irradiation by an 808 laser at a power density of 1.0 W cm^{-1}.

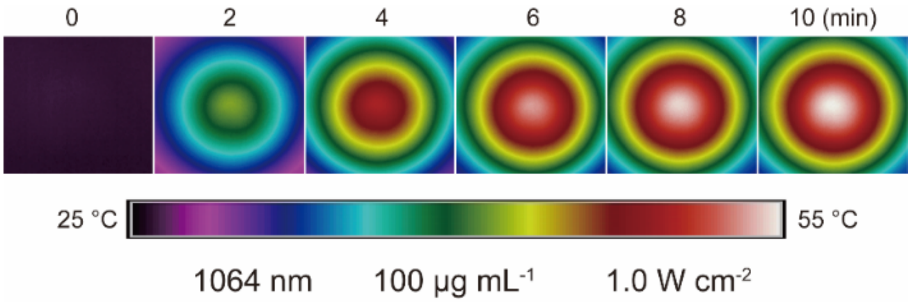

Figure S2. Thermal images of Nb_2C MXene (100 µg mL^{-1}) under irradiation by a 1064 laser at a power density of 1.0 W cm^{-1}.

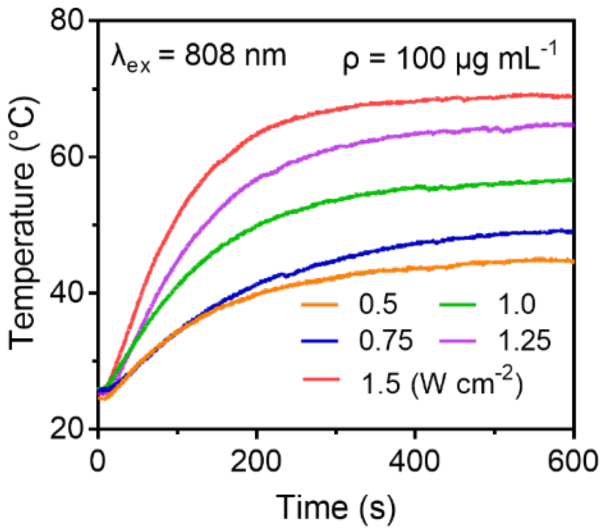

Figure S3. Photothermal profile of Nb_2C MXene (100 μg mL^{-1}) under irradiation by an 808 laser at different power densities.

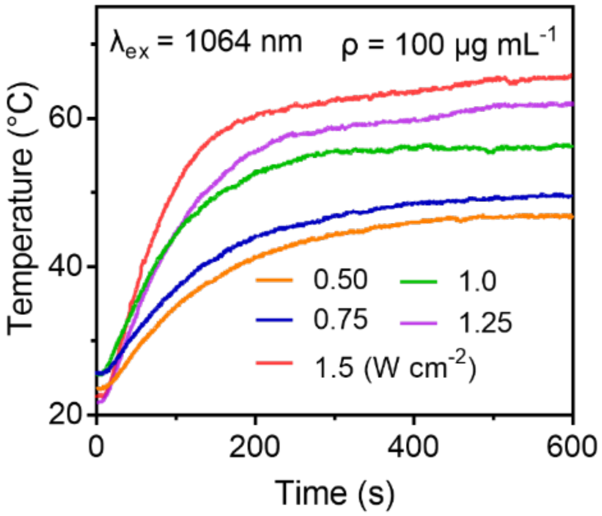

Figure S4. Photothermal profile of Nb_2C MXene (100 µg mL^{-1}) under irradiation by a 1064 laser at different power densities.

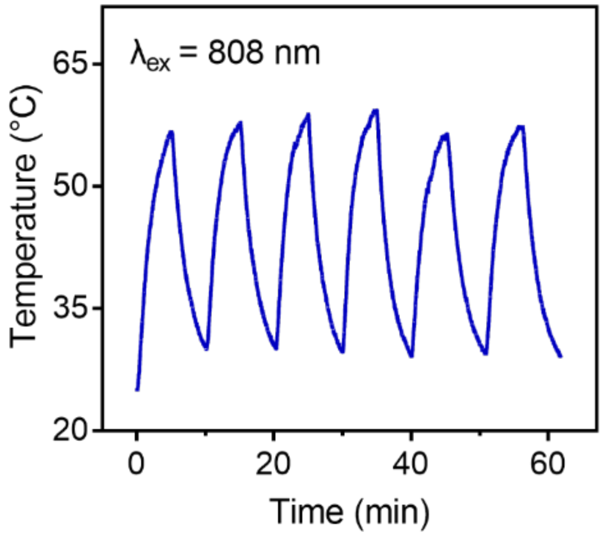

Figure S5. Photothermal curves of Nb_2C aqueous solution for six on/off irradiation cycles by an 808 nm laser (1 W cm^{-2}).

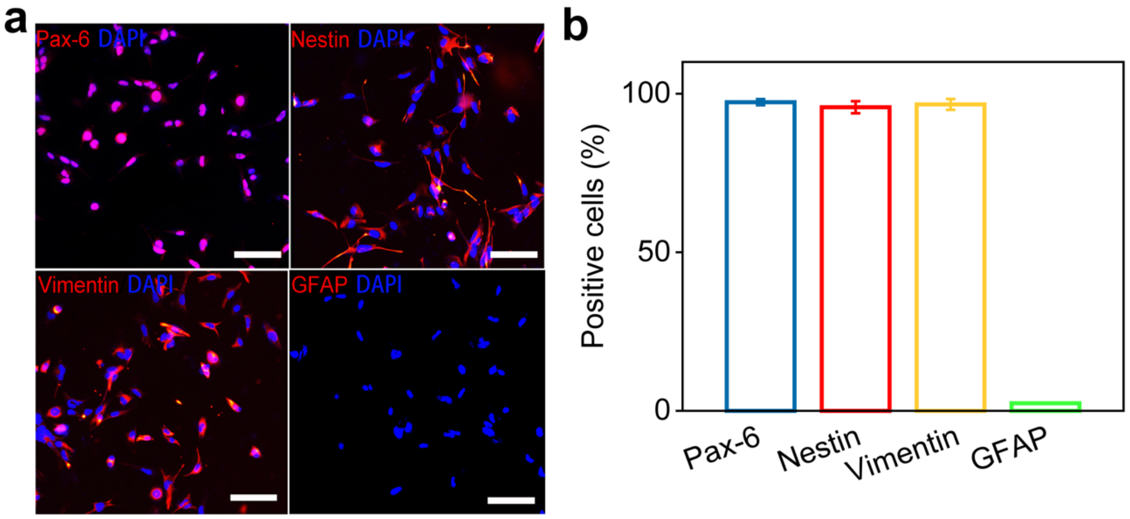

Figure. S6 Identification of specific markers of RPCs. a) Representative images of Pax-6, Nestin, Vimentin and GFAP-positive cells were taken after immunocytochemistry staining. Scale bars: 50 µm. b) Quantitative analysis of the positive ratio of Pax-6-, Nestin-, Vimentin-, and GFAP-expressed cells. n=6 independent experiments, data presented as mean ± SD.

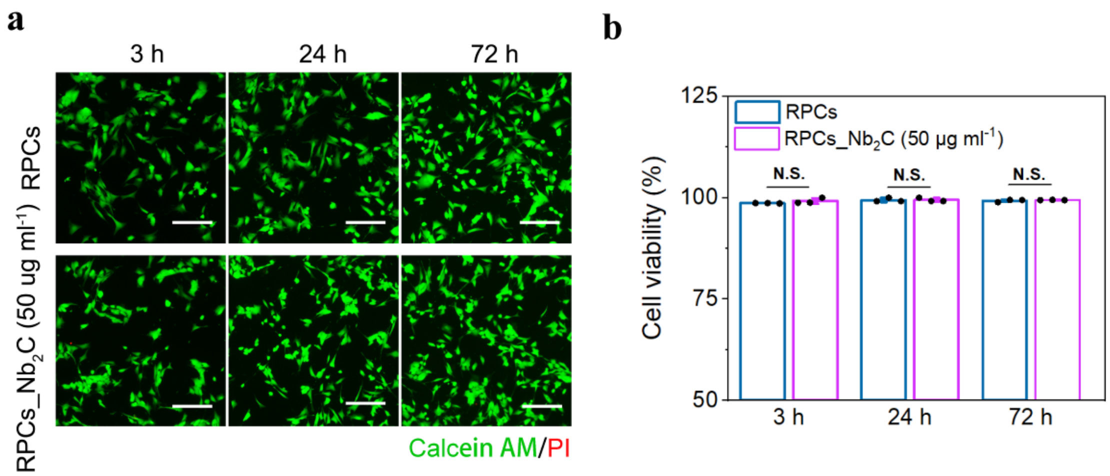

Figure S7. Effects of 2D Nb_2C MXene (50 μg ml^{-1}) on cell viability by a) live/dead staining after treatment for 3 h, 24 h and 72 h, and b) the survived cells were further calculated using Image J software. Living cells (green): calcein AM staining, dead cells (red): PI staining. Scale bars: 100 μm. n=3, not significant (N.S.) > 0.05 by two-tailed Student's t-test, data presented as mean ± SD.

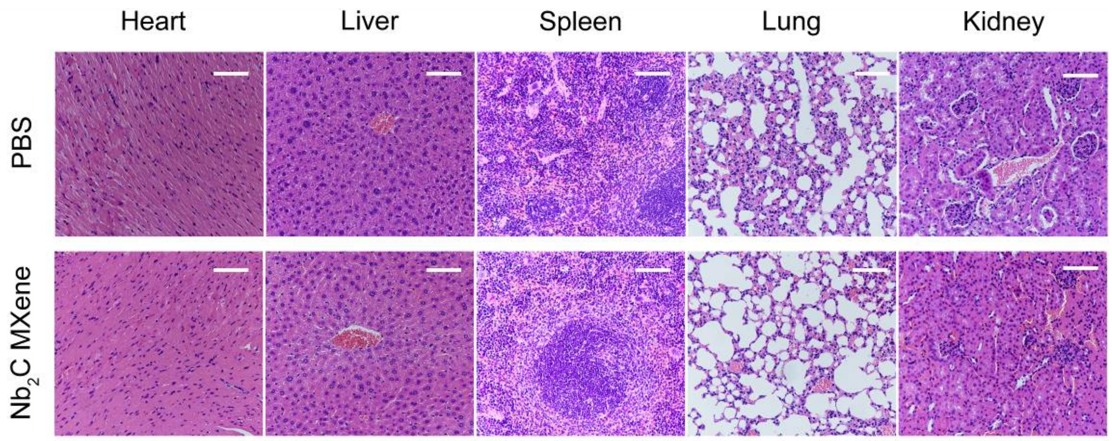

Figure S8. Representative H&E staining images of organic tissues, including heart, liver, spleen, lung and kidney were taken in Nb_2C MXene-injected healthy mice 2-weeks post-administration. Scale bars: 100 μm.

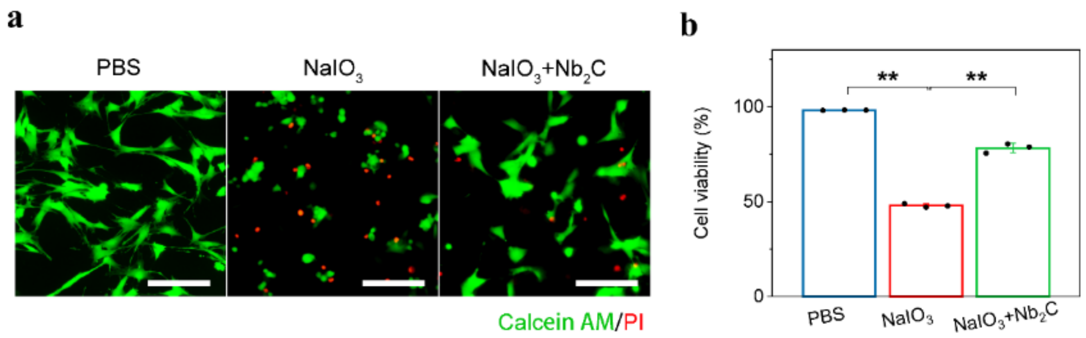

Figure S9. RPCs treated with PBS, $NaIO_3$ (10 mM), and $NaIO_3$ + Nb_2C under differentiation condition for 24 h, then a) representative live/dead RPCs were imaged, and b) the survived cells were further calculated using Image J software. Scale bars: 100 μm. n=3, one-way ANOVA with Bonferroni correction, $^{**}P < 0.01$, data presented as mean ± SD.

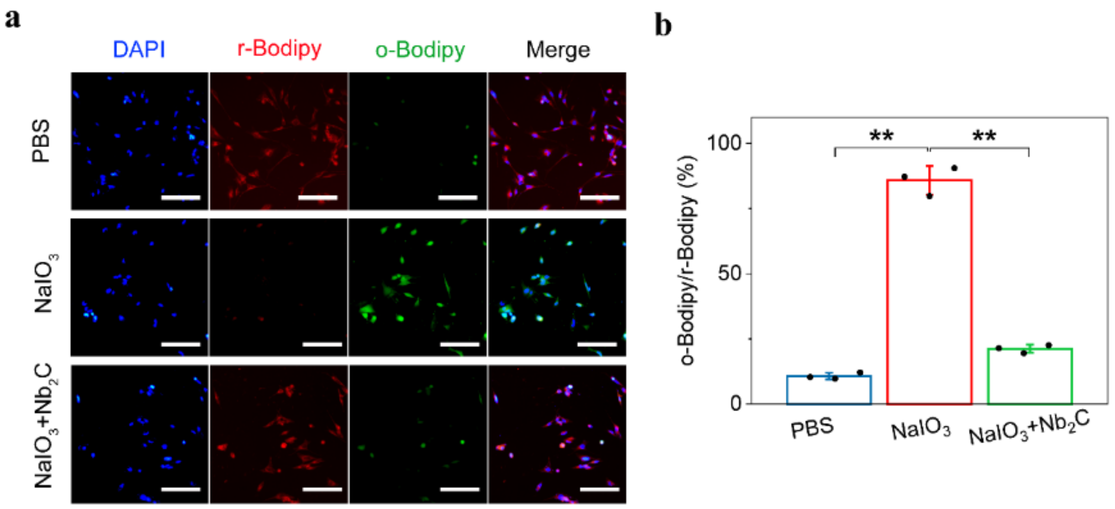

Figure S10. RPCs treated with PBS, $NaIO_3$, and $NaIO_3$ + Nb_2C MXene under differentiation condition for 24 h, then a) lipid peroxidation in RPCs was determined using C11-BODIPY probe and b) relative fluorescence intensity of oxidized-Bodipy was further calculated. Blue: cell nuclei, red: reduced-Bodipy, green: oxidized-Bodipy. Scale bars: 100 µm. n=3, one-way ANOVA with Bonferroni correction, $^{**}P < 0.01$, data presented as mean ± SD.

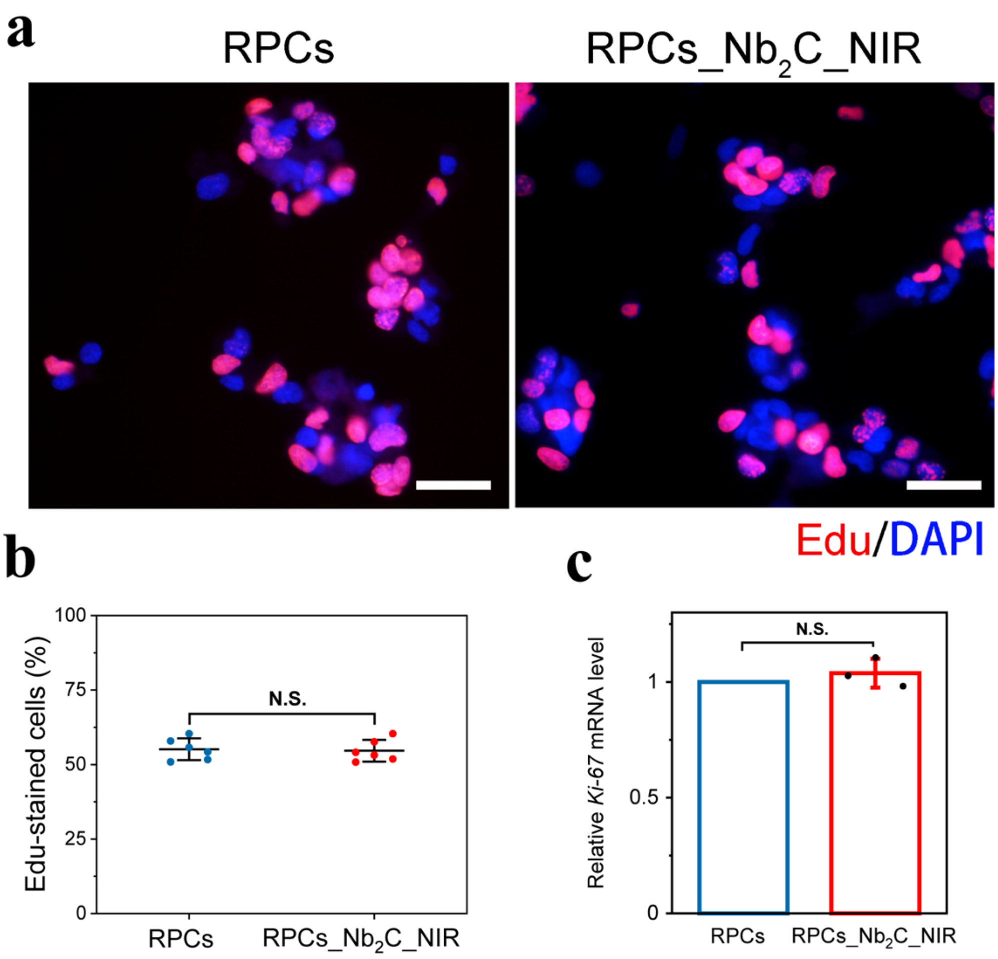

Figure S11. After 3 days of Nb_2C_NIR treatment under proliferation culture, a) proliferation capability of RPCs was subject to Edu-staining assay, and b) Edu-positive cell percentage was calculated by Image J software. Scale bars: 50 μm. n=6, not significant (N.S.) > 0.05 by two-tailed Student's t-test. c) Gene expression level of Ki-67 (a cell proliferation marker) was additionally detected. n=3, not significant (N.S.) > 0.05 by two-tailed Student's t-test, data presented as mean ± SD.

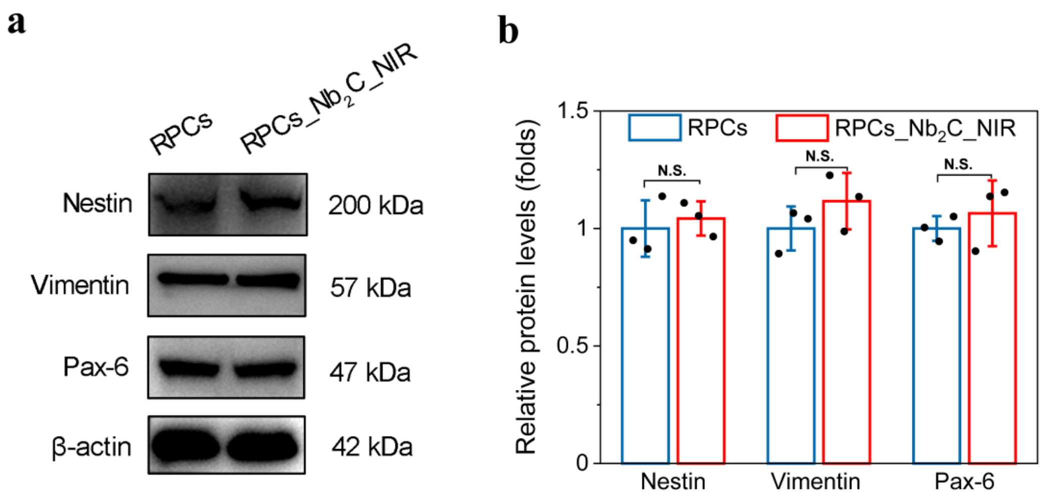

Figure S12. After 3 days of Nb_2C_NIR treatment under proliferation culture, stemness of RPCs was evaluated by detecting a) the protein expression levels and d) quantitative analysis of retinal progenitor-related markers (Nestin, Vimentin and Pax-6). n=3, not significant (N.S.) > 0.05 by two-tailed Student's t-test, data presented as mean ± SD.

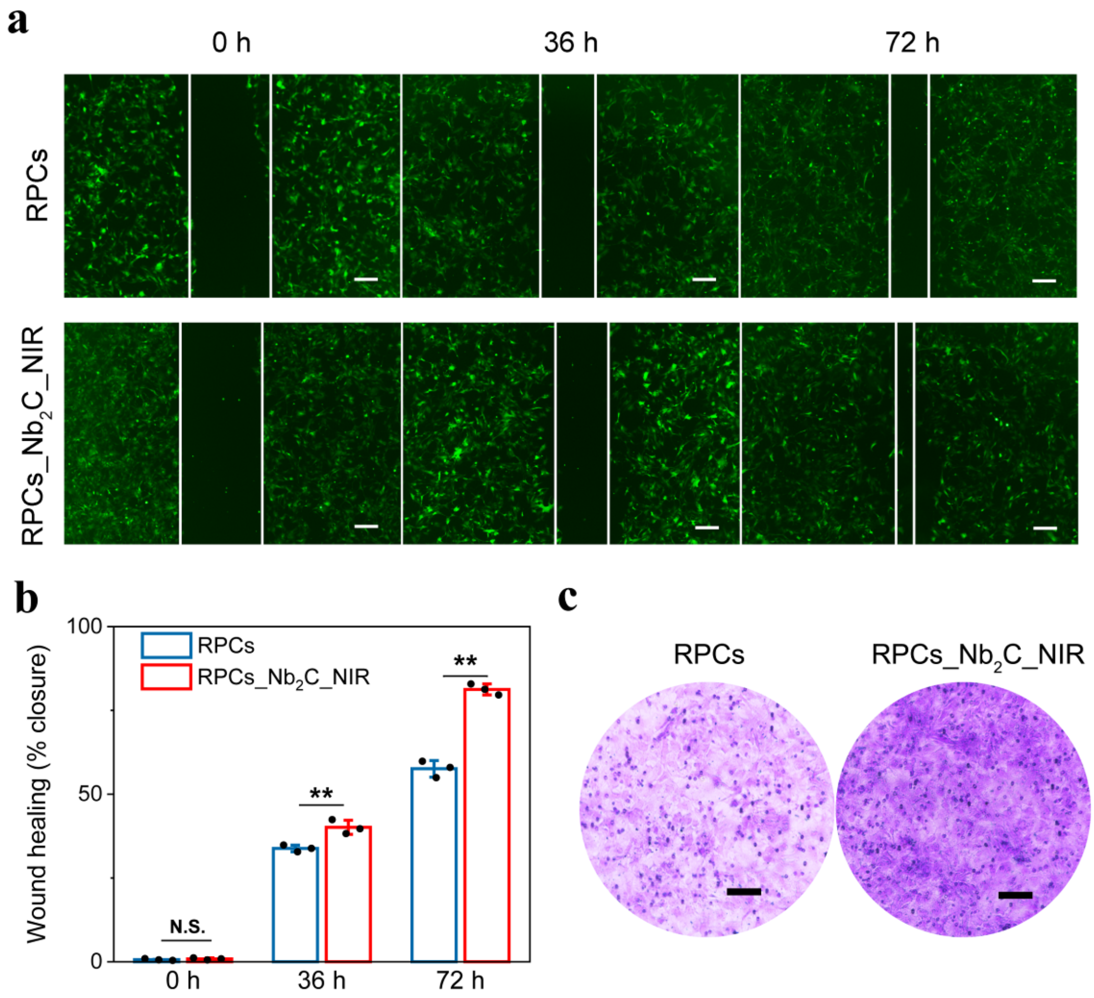

Figure S13. a) Representative imaging and b) quantitative analysis of cell horizontal migration across the wound areas (indicated between two white lines) after scratch for 36 h and 72 h. Scale bars: 100 µm. n=3, **P < 0.01, not significant (N.S.) > 0.05 by two-tailed Student's t-test. c) Transwell assay demonstrated the vertical migration of Nb_2C_NIR treated RPCs by 1% crystal violet staining. Scale bars: 100 µm, data presented as mean ± SD.

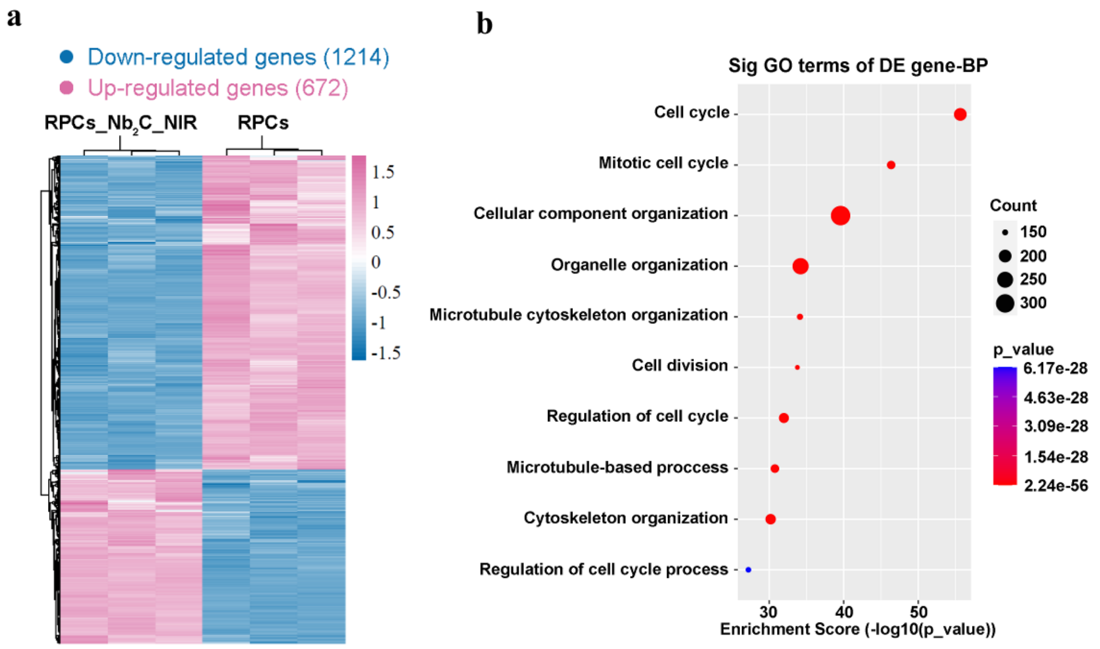

Figure S14. a) Heatmap showed differentially expressed genes across Nb_2C_NIR treated RPCs (RPCs_Nb_2C_NIR) against PBS-treated RPCs (RPCs). Red: upregulated genes, blue: downregulated genes. b) Top ten of significant GO terms by enrichment analysis of differentially downregulated genes involving biological process.

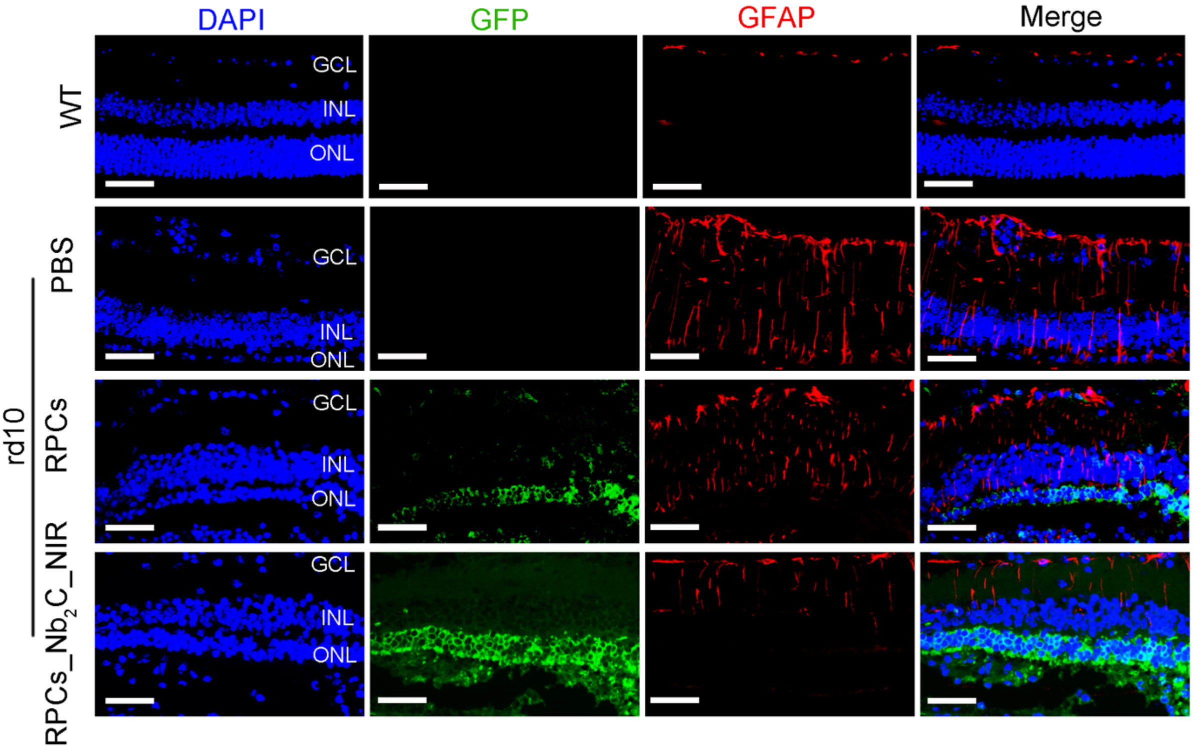

Figure. S15 Immunostaining of GFAP in WT and rd10 retina 8 weeks after transplantation of PBS, GFP^+ RPCs or GFP^+ RPCs_Nb_2C_NIR. Scale bars: 50 μm. n=6 independent experiments. GFAP (red): glial fibrillary acidic protein. WT: wild type. ONL: outer nuclear layer, INL: inner nuclear layer, GCL: ganglion cell layer.

S2. Supporting Tables S1~S2

Table S1. Primer sequences for qPCR analysis

Genes	Accession number	Forward (5-3′)	Reverse (5-3′)	Annealing temperature (°C)	Product size (base pairs)
Ki-67	X82786	cagtactcggaatgcagcaa	cagtcttcaggggctctgtc	60	170
IL-6	NM_031168.1	aggagtggctaaggaccaaga	ataacgcactaggtttgccga	60	100
MCP-1	NM_011333.3	acctgctgctactcattcacc	attccttcttggggtcagca	60	148
Caspase-3	NM_004346	catggaagcgaatcaatggact	ctgtaccagaccgagatgtca	60	139
β3-tubulin	NM_023279	cgagacctactgcatcgaca	cattgagctgaccagggaat	60	152
Rhodopsin	NM_145383	tcaccaccaccctctacaca	tgatccaggtgaagaccaca	60	216
PKC-α	NM_011101	cccattccagaaggagatga	ttcctgtcagcaagcatcac	60	212
GFAP	NM_010277	agaaaaccgcatcaccattc	tcacatcaccacgtccttgt	60	184
β-actin	NM_007393	agccatgtacgtagccatcc	ctctcagctgtggtggtgaa	60	152

Table S2. Primary antibodies used in western blot, immunocytochemistry (ICC) and/or immunofluorescence analysis

Antibodies	Animal	Vendor	Western blot	ICC	Immunofluorescence
β-actin	Mouse	Proteintech	Yes (1:5000)	No	No
PKC-α	Rabbit	Cell Signaling Technology	Yes (1:800)	Yes (1:200)	Yes (1:200)
β3-tubulin	Rabbit	Sigma-Aldrich	Yes (1:800)	Yes (1:200)	No
GFAP	Mouse	Sigma-Aldrich	Yes (1:800)	Yes (1:200)	Yes (1:200)
Rhodopsin	Mouse	Sigma-Aldrich	Yes (1:800)	Yes (1:200)	Yes (1:200)
Recoverin	Rabbit	Proteintech	No	No	Yes (1:200)
RBPMS	Rabbit	Proteintech	No	No	Yes (1:200)
Caspase-3	Mouse	Proteintech	Yes (1:800)	No	No
MCP-1	Mouse	Santa Cruz Biotechnology	Yes (1:800)	No	No
IL-6	Rabbit	Abcam	Yes (1:800)	No	No
CD68	Rabbit	Abcam	No	No	No
Nestin	Rabbit	Sigma-Aldrich	Yes (1:800)	Yes (1:200)	No
Vimentin	Rabbit	Sigma-Aldrich	Yes (1:800)	Yes (1:200)	No
Pax-6	Rabbit	Sigma-Aldrich	Yes (1:800)	Yes (1:200)	No
p-Erk	Rabbit	Proteintech	Yes (1:800)	No	No
Erk	Mouse	Proteintech	Yes (1:800)	No	No
p-Akt	Rabbit	Proteintech	Yes (1:800)	No	No
Akt	Rabbit	Abcam	Yes (1:800)	No	No
